# Sample Size Estimation in Veterinary Epidemiologic Research

**DOI:** 10.3389/fvets.2020.539573

**Published:** 2021-02-17

**Authors:** Mark A. Stevenson

**Affiliations:** Faculty of Veterinary and Agricultural Sciences, The University of Melbourne, Parkville, VIC, Australia

**Keywords:** sampling, epidemiiology, multilevel—hierarchical clustering, veterinary science, biostatistics

## Abstract

In the design of intervention and observational epidemiological studies sample size calculations are used to provide estimates of the minimum number of observations that need to be made to ensure that the stated objectives of a study are met. Justification of the number of subjects enrolled into a study and details of the assumptions and methodologies used to derive sample size estimates are now a mandatory component of grant application processes by funding agencies. Studies with insufficient numbers of study subjects run the risk of failing to identify differences among treatment or exposure groups when differences do, in fact, exist. Selection of a number of study subjects greater than that actually required results in a wastage of time and resources. In contrast to human epidemiological research, individual study subjects in a veterinary setting are almost always aggregated into hierarchical groups and, for this reason, sample size estimates calculated using formulae that assume data independence are not appropriate. This paper provides an overview of the reasons researchers might need to calculate an appropriate sample size in veterinary epidemiology and a summary of sample size calculation methods. Two approaches are presented for dealing with lack of data independence when calculating sample sizes: (1) inflation of crude sample size estimates using a design effect; and (2) simulation-based methods. The advantage of simulation methods is that appropriate sample sizes can be estimated for complex study designs for which formula-based methods are not available. A description of the methodological approach for simulation is described and a worked example provided.

## Introduction

In the design of intervention and observational epidemiological studies sample size calculations are used to provide estimates of the minimum number of observations that need to be made to ensure that the stated objectives of a study are met (1, 2). Peer reviewed journals require investigators to provide justification of the number of subjects enrolled into a study and details of the assumptions and methodologies used to derive sample size estimates are now a mandatory component of grant application processes (3). Studies lacking in justification of sample size run the risk of failing to identify differences among treatment or exposure groups if a difference in those groups actually exist (4). Selection of a number of study subjects greater than that actually required results in a wastage of time and resources (2).

Methods for sample size estimation vary depending on the type of study being carried out i.e., observational (non-experimental) or interventional (experimental). Formula-based approaches for sample size estimation are often preferred by investigators because: (1) they are relatively quick and simple to implement; (2) their widespread use makes peer review challenge less likely; and (3) the ability to use standard formulae goes hand in hand with “standard” study designs (i.e., randomized clinical trials, cross-sectional studies, case-control studies or cohort studies). Use of a standard study design implies the use of established approaches for data collection and analysis, again reducing the likelihood of challenge during peer review. In veterinary epidemiology the aggregation of animals into often several levels of hierarchy (e.g., cows within pens, pens within herds, herds within farms, and farms within regions) complicates sample size calculations due to lack of data independence arising from study subjects being aggregated into groups (e.g., pens, herds, farms, and regions). While modifications to standard sample size formulae are available, their flexibility to handle the range of real-world data situations is often limited.

The aim of this paper is to provide an overview of sample size estimation methods and their usage in applied veterinary epidemiological research. The structure of the paper is as follows. In the first section an overview of formula-based approaches for sample size estimation in epidemiological research is provided. In the second section, formula-based approaches for calculation of appropriate samples sizes for clustered data are presented. In the third and final section simulation-based approaches are presented as a means for estimating an appropriate sample size for hierarchical study designs for which formula-based methods are not available. Examples are provided throughout the paper to illustrate and support the concepts discussed. The supplementary material contains code allowing readers to reproduce the results presented in each of the examples using functions available in the contributed epiR package (5) in R (6).

## Formula-Based Approaches for Sample Size Estimation

In veterinary epidemiology sample size calculations are used during the design phase of a study to allow investigators to: (1) estimate a population parameter (e.g., the prevalence of disease); (2) test a hypothesis in an observational setting (using, for example, one of the three main observational study designs: cross-sectional, cohort or case-control); (3) test a hypothesis in an intervention setting (using a randomized clinical trial); and (4) achieve a specified level of confidence that an event will be detected if it is present at a specified design prevalence.

### Sample Size Calculations to Estimate a Population Parameter

A summary of formula-based methods for estimation of a population parameter, all assuming data independence, is provided in Tables 1, 2. Methods are defined for continuous and binary outcomes with different calculation methods dependent on the proposed sampling design: simple random, stratified random, one-stage cluster and two-stage cluster designs. For continuous outcomes the analyst needs to provide an estimate of the mean of the outcome of interest and its expected variability. For binary outcomes only an estimate of the expected population proportion is required, given the variance of a proportion *P* equals *P* × [1 − *P*] (8). In addition to specifying the required level of confidence in the population parameter estimate (usually 95%) one needs to specify the desired maximum tolerable error. The maximum tolerable error is the difference between the true population parameter and the estimate of the true population parameter derived from sampling. In each of the formula-based approaches listed in Tables 1, 2 tolerable error is expressed in relative (as opposed to absolute) terms. If one assumes that the true population prevalence of disease is 0.40 and a desired relative tolerable error of 0.10 with 95% confidence is required, this means the calculation will return the required number of subjects to be 95% certain that the prevalence estimate from the study will be anywhere between 0.40 ± (0.10 × 0.40) that is, from 0.36 to 0.44. Some sample size formulae and/or software packages require maximum tolerable error to be expressed in absolute terms (that is, 0.04 for the example cited above). Analysts should take care to ensure that there is no ambiguity around the input format for tolerable error when using a published formula or software package since the distinction between absolute and relative error is often not clear in either the formula documentation or the graphic user interface, in the case of computer software. Similarly, when making a statement of the criteria used for sample size calculations when reporting the results of a study, care should be taken to ensure that the “relative” or “absolute” qualifier is used when referring to tolerable error.

**Table 1:** Information required to estimate a sample size for each of the common sampling designs, binary or continuous population parameters.

**Outcome variable**	**Sampling design**	**Arguments**	**References**
Continuous	Simple random	Total number of individual listing units in the population, the relative variance of the continuous variable to be estimated (i.e., the variance divided by the mean squared).	(7) pp. 74, Equation 3.14
Continuous	Stratified random	Total number of individual listing units in each strata, the expected means of the continuous variable to be estimated for each strata, the expected variances of the continuous variable to be estimated for each strata.	(7) pp. 176, Equation 6.25
Continuous	One stage cluster	Total number of clusters in the population, the population mean of the continuous variable to be estimated, the population variance of the continuous variable to be estimated.	(7) pp. 255, Box 9.4
Continuous	Two stage cluster	Number of individual listing units to be sampled from each cluster, the total number of clusters in the population and the number of individual listing units in each cluster, the mean of the continuous variable to be estimated at the first and second stage of sampling, the variance of the continuous variable to be estimated at the first and second stage of sampling.	(7) pp. 289, Equation 10.6
Binary	Simple random sampling	Total number of individual listing units in the population, the expected proportion of individual listing units with the outcome of interest.	(7) pp. 74, Equation 3.16
Binary	Stratified random	Total number of individual listing units in each strata, the expected proportion of individual listing units with the outcome of interest for each strata.	(7) pp. 176, Equation 6.23
Binary	One stage cluster	Total number of clusters in the population, the mean of the proportion of individual listing units in each cluster with the outcome of interest, the variance of the proportion of individual listing units in each cluster with the outcome of interest.	(7) pp. 255 Box 9.4
Binary	Two stage cluster	Number of individual listing units to be sampled from each cluster, the total number of clusters in the population and the number of individual listing units within each cluster, the mean of the denominator variable used to calculate the unknown population proportion at the first and second stage of sampling, the variance of the denominator variable used to calculate the unknown population proportion at the first and second stage of sampling, the variance of the numerator variable used to calculate the unknown population proportion at the first and second stage of sampling, the covariance of the unknown population proportion at the first and second stage of sampling.	(7) pp. 289, Equation 10.7

**Table 2:** Formulae to estimate a sample size for each of the common sampling designs, binary or continuous population parameters.

**Outcome variable**	**Sampling design**	**Formula**	**Arguments**
Continuous	Simple random	$n\geq \frac{{z_{1-\left(\alpha /2\right)}}^{2}N\;V_{x}^{2}\;}{{z_{1-\left(\alpha /2\right)}}^{2}\;V_{x}^{2}+\left(N-1\right)\;\epsilon_{r}^{2}}$	*n* = the number of subjects in the sample. *z*_1−(α/2)_ = value from the standard normal curve corresponding to the desired level of confidence. Use *z*_1−(α/2)_ = 1.96 for 95% (two-sided) confidence. *N* = the population size. *V*_*x*_ = the relative variance (the variance divided by the mean squared). ϵ_*r*_ = the relative error.
Continuous	Stratified random	$n\geq \frac{{z_{1-\left(\alpha /2\right)}}^{2}\times \frac{N}{1+\;\gamma }\times V_{x}^{2}}{N\epsilon_{r}^{2}+\;{z_{1-\left(\alpha /2\right)}}^{2}\times \frac{V_{x}^{2}}{1+\;\gamma }}$	*N* = the number of subjects in the sample. *z*_1−(α/2)_ = value from the standard normal curve corresponding to the desired level of confidence. Use *z*_1−(α/2)_ = 1.96 for 95% (two-sided) confidence. *N* = the population size. γ = between strata variance $\sigma_{bx}^{2}$ divided by the within strata variance. $\sigma_{wx}^{2}$. $V_{x}^{2}=$ the relative variance (the variance divided by the mean squared). ϵ_*r*_ = the relative error.
Continuous	One stage cluster	$m=\;\frac{{z_{1-\left(\alpha /2\right)}}^{2}MV_{1x}^{2}}{{z_{1-\left(\alpha /2\right)}}^{2}V_{1x}^{2}+\left(M-1\right)\epsilon_{r}^{2}}$ $V_{1x}^{2}=\;\frac{\sigma_{1x}^{2}}{{\bar{X}}^{2}}$ $\sigma_{1x}=\;\frac{\sum_{i=1}^{M}\left(X_{i}-\bar{X}\right)\left(Y_{i}-\bar{Y}\right)}{M}$	*m* = the number of clusters in the sample *z*_1−(α/2)_ = value from the standard normal curve corresponding to the desired level of confidence. Use *z*_1−(α/2)_ = 1.96 for 95% (two-sided) confidence. *M* = the number of clusters in the population. ϵ_*r*_ = the relative error. $\sigma_{1x\;}^{2}=$ the first stage variance components. $\bar{X}=$ mean level of *X* per cluster. *X*_*i*_ = level of the *i*th value of characteristic *X*.
Continuous	Two stage cluster	$m=\;\frac{\left(\frac{\sigma_{1x}^{2}}{{\bar{X}}^{2}}\right)\;\times \;\left(\frac{M}{M-1}\right)+\left(\;\frac{1}{\bar{n}}\right)\times \;\left(\frac{\sigma_{2x}^{2}}{{\bar{X}}^{2}}\right)\;\times \;\left(\frac{\bar{N}-\bar{n}}{\bar{N}-1}\right)}{\frac{\varepsilon_{r}^{2}}{{z_{1-\left(\alpha /2\right)}}^{2}}+\;\frac{\sigma_{1x}^{2}}{{\bar{X}}^{2}\left(M-1\right)}}$	*M* = the number of clusters in the sample. $\sigma_{1x\;}^{2}=$ the first stage variance components. $\bar{X}=$ mean level of *X* per cluster. *M* = the number of clusters in the population. $\bar{n}=$ the number of listing units to be sampled from each cluster. $\sigma_{2x\;}^{2}=$ the second stage variance components. $\bar{N}=$ the number of listing units in each cluster. ϵ_*r*_ = the relative error. *z*_1−(α/2)_ = value from the standard normal curve corresponding to the desired level of confidence. Use *z*_1−(α/2)_ = 1.96 for 95% (two-sided) confidence.
Binary	Simple random sampling	$n\geq \frac{{z_{1-\left(\alpha /2\right)}}^{2}N\;P_{y}\;\left(1-P_{y}\right)}{\left[\left(N-1\right)\epsilon_{r}^{2}P_{y}^{2}\right]+\;{z_{1-\left(\alpha /2\right)}}^{2}\;P_{y}\left(1-P_{y}\right)}$	*N* = the number of subjects in the sample. *N* = the population size *P*_*y*_ = the estimated population prevalence. ϵ_*r*_ = the relative error. *z*_1−(α/2)_ = value from the standard normal curve corresponding to the desired level of confidence. Use *z*_1−(α/2)_ = 1.96 for 95% (two-sided) confidence.
Binary	Stratified random	$n\geq \frac{\left(\frac{{z_{1-\left(\alpha /2\right)}}^{2}}{N^{2}}\right)\sum_{h=1}^{L}\frac{N_{h}^{2}\;P_{hy}\left(1-P_{hy}\right)}{\pi_{h}P_{y}^{2}}}{\epsilon_{r}^{2}+\;\left(\frac{{z_{1-\left(\alpha /2\right)}}^{2}}{N^{2}}\right)\left(\sum_{h=1}^{L}\frac{N_{h}P_{hy}\left(1-P_{hy}\right)}{P_{y}^{2}}\right)}$ $\pi_{h}=\;\frac{n_{h}}{n}$	*N* = the number of subjects in the sample *z*_1−(α/2)_ = value from the standard normal curve corresponding to the desired level of confidence. Use *z*_1−(α/2)_ = 1.96 for 95% (two-sided) confidence. *N* = the population size. *L* = the number of strata. *N*_*h*_ = the population size in the *h*th strata. *P*_*hy*_ = the estimated population prevalence in the *h*th strata. *P*_*y*_ = the estimated population prevalence. ϵ_*r*_ = the relative error. π_*h*_ = the fraction of samples allocated to strata *h* (decided in advance).
Binary	One stage cluster	When the number of listing units to be sampled per cluster is the same: *D* = 1+(*b*−1)ρ When the number of listing units to be sampled per cluster varies: $D=1+\left\{\left(CV^{2}+1\right)\;\bar{b}-1\right\}\rho$ $n_{c}\geq \;\frac{{z_{1-\left(\alpha /2\right)}}^{2}\;P_{y}\;\left(1-P_{y}\right)D}{{\left(P_{y}\;\epsilon_{r}\right)}^{2}\bar{b}}$	*D* = the design effect. *b* = the number of listing units to be sampled from each cluster. ρ= the intracluster correlation coefficient. *CV* = the coefficient of variation of the number of listing units to be sampled from each cluster. $\bar{b}=$ the average number of listing units to be sampled from each cluster. *N*_*c*_ = the number of primary sampling units (clusters) to be sampled. *z*_1−(α/2)_ = value from the standard normal curve corresponding to the desired level of confidence. Use *z*_1−(α/2)_ = 1.96 for 95% (two-sided) confidence. *P*_*y*_ = the estimated population prevalence ϵ_*r*_ = the relative error.
Binary	Two stage cluster	$m=\;\frac{\left(\frac{\sigma_{1R}^{2}}{{\bar{X}}^{2}}\right)\;\times \;\left(\frac{M}{M-1}\right)+\left(\;\frac{1}{\bar{n}}\right)\times \;\left(\frac{\sigma_{2R}^{2}}{{\bar{X}}^{2}}\right)\;\times \;\left(\frac{\bar{N}-\;\bar{n}}{\bar{N}-1}\right)}{\frac{\varepsilon_{r}^{2}}{{z_{1-\left(\alpha /2\right)}}^{2}}+\;\frac{\sigma_{1R}^{2}}{{\bar{X}}^{2}\left(M-1\right)}}$	*M* = the number of clusters in the sample. $\sigma_{1R\;}^{2}=$ the first stage variance components. $\bar{X}=$ the mean level of characteristic *X* per listing unit. *M* = the number of clusters in the population. $\bar{n}=$ the number of listing units to be sampled from each cluster. $\sigma_{2R\;}^{2}=$ the first stage variance components. $\bar{X}$ = the mean level of characteristic *X* per cluster. $\bar{N}=$ the average number of listing units per cluster in the population. ϵ_*r*_ = the relative error. *z*_1−(α/2)_ = value from the standard normal curve corresponding to the desired level of confidence. Use *z*_1−(α/2)_ = 1.96 for 95% (two-sided) confidence.

In the absence of prior knowledge of the event prevalence in a population a conservative sample size estimate can be made assuming event prevalence is 0.5, since the variance of a prevalence (that is, *P* × [*P* − 1]) is greatest when *P* = 0.5 and the absolute tolerable error and level of confidence remains fixed (8).

A worked example of a sample size calculation to estimate a prevalence using simple random sampling is shown in Box 1.

Box 1 **The expected seroprevalence of brucellosis in a population of cattle is thought to be in the order of 15%. How many cattle need to be sampled and tested to be 95% certain that our seroprevalence estimate is within 20% (i.e., 0.20 × 0.15 = 0.03, 3%) of the true population value, assuming use of a test with perfect sensitivity and specificity? This formula requires the population size to be specified so we set *N* to a large number, 1,000,000:**

$n\geq \frac{{z_{1-\left(\alpha /2\right)}}^{2}\;N\;P_{y}\;\left(1-\;P_{y}\right)}{{\left[\left(N-1\right)\epsilon_{r}^{2}\;P_{y}^{2}\right]+\;z_{1-\left(\alpha /2\right)}}^{2}P_{y}\left(1-\;P_{y}\right)}$

$\;n\geq \frac{1.96^{2}\;\times 1,000,000\;\times 0.15\left(1-\;0.15\right)\;}{{\left[\left(1,000,000-1\right)0.20^{2}\;0.15^{2}\right]+\;1.96}^{2}\;\times \;0.15\;\times \;\left(1-\;0.15\right)}$

$\;n\geq \frac{489,804\;}{900.489}$

      *n* ≥ 545

To be 95% confident that our estimate of brucellosis seroprevalence is within 20% of the true population value (i.e., a relative error of 0.20) 545 cattle should be sampled.

With stratified sampling the sampling frame is divided into groups (strata) and a random sample is taken from each stratum. When the variation of the outcome of interest within each stratum is small relative to the variation between strata, stratified random sampling returns a more precise estimate of the population parameter compared with simple random sampling.

### Sample Size Calculations to Test a Hypothesis Using an Observational Study Design

Details of the formula-based methods to estimate a sample size for each of the main observational study (i.e., cross-sectional, case-control, and cohort studies) are provided in Tables 3, 4. Again, these formulae all assume that data are independent. Note that the sample size formulae for cross-sectional studies, cohort studies using count data and cohort studies using time at risk require the analyst to provide an estimate of prevalence, incidence risk and incidence rate (respectively) for both risk factor exposed and unexposed groups. Box 2 provides a worked example for a prospective cohort study, with a fixed follow-up time.

**Table 3:** Information required to estimate a sample size for each of the common observational epidemiological study designs.

**Study design**	**Arguments**	**References**
Cross-sectional	The expected prevalence of the outcome among the exposed, the expected prevalence of the outcome among in the unexposed, the required study power, the ratio of the number of exposed subjects to the number of unexposed subjects, sided test.	(9) pp. 313, Equation 8.14
Case-control	The expected odds ratio, the prevalence of exposure among controls, the required study power, the ratio of the number of control subjects to the number of case subjects, sided test.	(10)
Cohort, count data	The expected outcome incidence risk among the exposed, the expected outcome incidence risk among the unexposed, the required study power, the ratio of the number of exposed subjects to the number of unexposed subjects, sided test.	(9) pp. 313, Equation 8.14
Cohort, time at risk	The expected outcome incidence rate among the exposed, the expected outcome incidence rate among the unexposed, the required study power, the ratio of the number of exposed subjects to the number of unexposed subjects, sided test.	(11)

**Table 4:** Formulae to estimate a sample size for each of the common observational epidemiological study designs.

**Study design**	**Formula**	**Arguments**
Cross-sectional	$n\geq \frac{r+1}{r{\left(\lambda -1\right)}^{2}\pi^{2}}\;{\left[z_{1-\left(\alpha /2\right)}\;\sqrt{\left(r+1\right)p_{c}\left(1-\;p_{c}\right)}+\;z_{1-\beta }\;\sqrt{\lambda \pi \left(1-\;\lambda \pi \right)+r\pi \left(1-\;\pi \right)}\right]}^{2}$	*n* = the number of subjects in the sample. *r* = the anticipated number of subjects in the exposed group divided by the anticipated number of subjects in the unexposed group. λ = the expected prevalence ratio. π = the expected prevalence of the outcome among the non-exposed. *z*_1−(α/2)_ = value from the standard normal curve corresponding to the desired level of confidence. Use *z*_1−(α/2)_ = 1.96 for 95% (two-sided) confidence. *p*_*c*_ = the common prevalence over exposed and unexposed groups.*z*_1−β_ = value from the standard normal curve corresponding to the desired study power. Use *z*_1−β_ = −0.84 for 80% power.
Case-control	$p_{c}*=\;\frac{p_{0}}{r+1}\;\left(\frac{r\lambda }{1+\left(\lambda -1\right)p_{0}}+1\right)$ $n\geq \;\frac{\left(r+1\right){\left(1+\left(\lambda -1\right)p_{0}\right)}^{2}}{rp_{0}^{2}{\left(p_{0}-1\right)}^{2}{\left(\lambda -1\right)}^{2}}\;{\left[z_{1-\left(\alpha /2\right)}\;\sqrt{\left(r+1\right)p_{c}*\left(1-\;p_{c}*\right)}+\;z_{1-\beta }\;\sqrt{\frac{\lambda p_{0}\left(1-p_{0}\right)}{{\left[1+\left(\lambda -1\right)p_{0}\right]}^{2}}+rp_{0}\left(1-p_{0}\right)}\right]}^{2}$	*n* = the number of subjects in the sample.*p*_0_ = the expected prevalence of exposure among the controls. *r* = anticipated number of subjects in the control group divided by the anticipated number of subjects in the case group. λ = the expected odds ratio. *z*_1−(α/2)_ = value from the standard normal curve corresponding to the desired level of confidence. Use *z*_1−(α/2)_ = 1.96 for 95% (two-sided) confidence. *z*_1−β_ = value from the standard normal curve corresponding to the desired study power. Use *z*_1−β_ = 0.84 for 80% power.
Cohort, count data	$n\geq \frac{r+1}{r{\left(\lambda -1\right)}^{2}\pi^{2}}\;{\left[z_{1-\left(\alpha /2\right)}\;\sqrt{\left(r+1\right)p_{c}\left(1-\;p_{c}\right)}+\;z_{1-\beta }\;\sqrt{\lambda \pi \left(1-\;\lambda \pi \right)+r\pi \left(1-\;\pi \right)}\right]}^{2}$	*n* = the number of subjects in the sample. *r* = the anticipated number of subjects in the exposed group divided by the anticipated number of subjects in the unexposed group. λ = the expected incidence risk ratio. π = the expected prevalence of the outcome among the non-exposed. *z*_1−(α/2)_ = value from the standard normal curve corresponding to the desired level of confidence. Use *z*_1−(α/2)_ = 1.96 for 95% (two-sided) confidence. *p*_*c*_ = the common prevalence over exposed and unexposed groups.*z*_1−β_ = value from the standard normal curve corresponding to the desired study power. Use *z*_1−β_ = 0.84 for 80% power.
Cohort, time at risk	$\lambda_{0}^{{\;}^{\prime }}=\;\frac{\lambda_{0}^{3}FT}{\lambda_{0}\;FT-1+\;{exp}^{\left(-\lambda_{0}FT\right)}}$ $\lambda_{1}^{{\;}^{\prime }}=\;\frac{\lambda_{10}^{3}FT}{\lambda_{1}\;FT-1+\;{exp}^{\left(-\lambda_{1}FT\right)}}$ ${\bar{\lambda }}^{{\;}^{\prime }}=\;\frac{{\bar{\lambda }}^{3}\;FT}{\bar{\lambda }\;FT-1+\;{exp}^{\left(-\bar{\lambda }FT\right)}}$ *n*_*A*_ = *rn*_*B*_ $n_{A}\geq \frac{{\left(z_{1-\left(\alpha /2\right)}\;\sqrt{\left(1+r\right){\bar{\lambda }}^{{\;}^{\prime }}}+\;z_{1-\;\beta }\;\sqrt{\left(r\;\times \;\lambda_{1}^{{\;}^{\prime }}+\;\lambda_{0}^{{\;}^{\prime }}\right)}\right)\;}^{2}}{{r\left(\lambda_{1}^{{\;}^{\prime }}-\;\lambda_{0}^{{\;}^{\prime }}\right)}^{2}}$	*n*_*A*_ = the number of subjects in the sample. λ_0_ = the expected incidence rate among the unexposed.λ_1_ = the expected incidence rate among the exposed.$\bar{\lambda }=\left(\lambda_{0}+\;\lambda_{1}\right)\;/\;2$ *FT* = the expected follow-up period for the study. *r* = anticipated number of subjects in the exposed group divided by the anticipated number of subjects in the unexposed group. *z*_1−(α/2)_ = value from the standard normal curve corresponding to the desired level of confidence. Use *z*_1−(α/2)_ = 1.96 for 95% (two-sided) confidence. *z*_1−β_ = value from the standard normal curve corresponding to the desired study power. Use *z*_1−β_ = 0.84 for 80% power.

Box 2 **A prospective cohort study of dry food diets and feline lower urinary tract disease (FLUTD) in mature male cats is planned. A sample of cats will be selected at random from the population and owners who agree to participate in the study will be asked to complete a questionnaire at the time of enrolment. Cats enrolled into the study will be followed for at least 5 years to identify incident cases of FLUTD. The investigators would like to be 0.80 certain of being able to detect when the risk ratio of FLUTD is 1.4 for cats habitually fed a dry food diet, using a 0.05 significance test. Previous evidence suggests that the incidence risk of FLUTD in cats not on a dry food (i.e., “other”) diet is around 50 per 1000 per year. Assuming equal numbers of cats on dry food and other diets are sampled, how many cats should be enrolled into the study?**

$\lambda_{0}^{{\;}^{\prime }}=\;\frac{\lambda_{0}^{3}\;FT}{\lambda_{0}\;FT-1+\;{exp}^{\left(-\lambda_{0}FT\right)}}$ $\lambda_{1}^{{\;}^{\prime }}=\;\frac{\lambda_{1}^{3}\;FT}{\lambda_{1}\;FT-1+\;{exp}^{\left(-\lambda_{1}FT\right)}}$ ${\bar{\lambda }}^{{\;}^{\prime }}=\;\frac{{\bar{\lambda }}^{3}\;FT}{\bar{\lambda }\;FT-1+{exp}^{\left(-\bar{\lambda }FT\right)}}$

${\;n}_{A}\geq \frac{{\left(z_{1-\left(\alpha /2\right)}\;\sqrt{\left(1+r\right){\bar{\lambda }}^{{\;}^{\prime }}}+\;z_{1-\beta }\;\sqrt{\left(r\;\times \;\lambda_{1}^{{\;}^{\prime }}+\;\lambda_{0}^{{\;}^{\prime }}\right)}\right)\;}^{2}}{{r\left(\lambda_{1}^{{\;}^{\prime }}-\;\lambda_{0}^{{\;}^{\prime }}\right)}^{2}}\;$

${\;n}_{A}\geq \frac{{\left(1.96\;\sqrt{\left(1+1\right)0.0264^{2}}+\;0.84\;\sqrt{\left(1\;\times \;0.313+\;0.0217\right)}\right)\;}^{2}}{{1\left(0.07-\;0.05\right)}^{2}}\;$

$\;n_{A}\geq \frac{{\left(0.4509+0.1935\right)}^{2}}{0.0004}\;$

      *n*_*A*_ ≥ 1040

A total of 2,080 male cats need to be sampled to meet the requirements of the study (1,040 cats habitually fed dry food and 1,040 cats habitually fed “other” diet types).

In contrast to sample size formulae for cross-sectional and cohort studies, the sample size formula for case-control studies requires provision of an estimate of the prevalence of exposure amongst controls (Box 3). An additional consideration when estimating an appropriate sample size for a case-control study is specification of the design – either matched or unmatched (12). The process of matching provides a means for controlling for the effect of a known confounder with the added benefit of an increase in statistical efficiency (12, 13).

Box 3 **A case-control study of the association between white pigmentation around the eyes and ocular squamous cell carcinoma in Hereford cattle is planned. A sample of cattle with newly diagnosed squamous cell carcinoma will be compared for white pigmentation around the eyes with a sample of controls. Assuming an equal number of cases and controls, how many study subjects are required to detect an odds ratio of 2.0 with 0.80 power using a two-sided 0.05 test? Previous surveys have shown that around 0.30 of Hereford cattle without squamous cell carcinoma have white pigmentation around the eyes.**

$n\geq \;\frac{\left(r+1\right){\left(1+\left(\lambda -1\right)p_{0}\right)}^{2}}{rp_{0}^{2}{\left(p_{0}-1\right)}^{2}{\left(\lambda -1\right)}^{2}}\;{\left[z_{1-\left(\alpha /2\right)}\;\sqrt{\left(r+1\right)p_{c}^{*}\left(1-\;p_{c}^{*}\right)}+\;z_{\beta }\;\sqrt{\frac{\lambda p_{0}\left(1-p_{0}\right)}{{\left[1+\left(\lambda -1\right)p_{0}\right]}^{2}}+rp_{0}\left(1-p_{0}\right)}\right]}^{2}$

$\;n\geq \;\frac{\left(1+1\right){\left(1+\left(2-1\right)0.3\right)}^{2}}{1\;\times 0.3^{2}{\left(0.3-1\right)}^{2}{\left(2-1\right)}^{2}}\;{\left[1.96\;\sqrt{\left(1+1\right)0.38\left(1-\;0.38\right)}+\;0.84\;\sqrt{\frac{2\;\times 0.3\left(1-0.3\right)}{{\left[1+\left(2-1\right)0.3\right]}^{2}}+1\;\times 0.3\left(1-0.3\right)}\right]}^{2}$

$\;n\geq \;\frac{3.38}{0.0441}\;{\left[1.346+0.569\right]}^{2}$

     *n* ≥ 282

If the true odds for squamous cell carcinoma in exposed subjects relative to unexposed subjects is 2.0, we will need to enrol 141 cases and 141 controls (282 cattle in total) to reject the null hypothesis that the odds ratio equals one with probability (power) 0.80. The Type I error probability associated with this test of this null hypothesis is 0.05.

### Sample Size Calculations to Test a Hypothesis Using a Randomized Clinical Trial

A superiority trial is a study in which the aim is to show that a treatment intervention provides a better therapeutic outcome than a known reference (often a placebo) and the statistical procedure to provide this evidence is called a superiority test (14).

**Figure 1:**
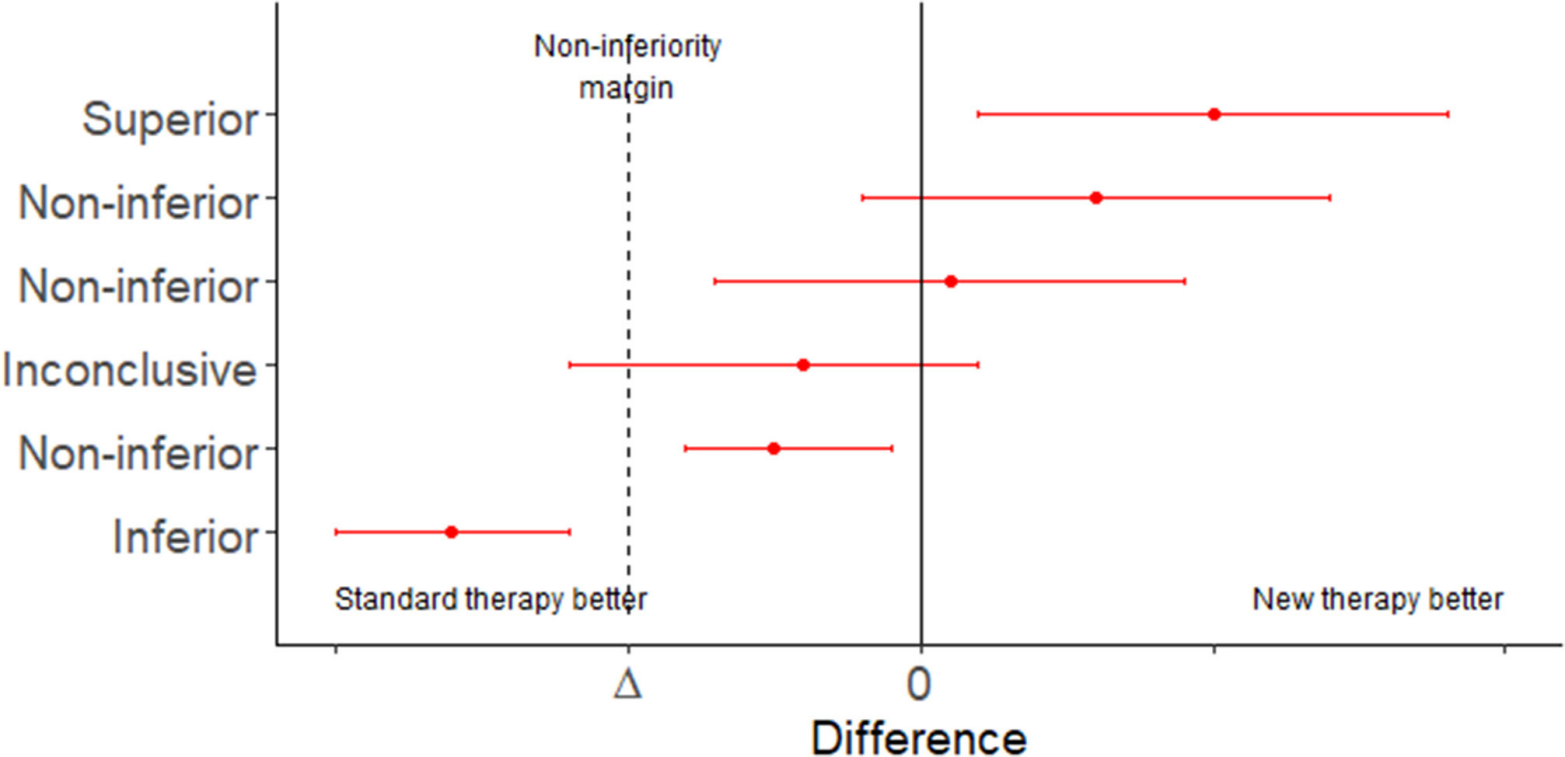
Error bar plot showing the possible conclusions to be drawn from a non-inferiority trial. Adapted from Head et al. (18).

In situations where an established treatment already exists a study comparing a new treatment to a placebo (effectively, no treatment) will be unethical. In this situation interest lies in determining if the new treatment is: (1) either the same as, or better than, an established treatment using a non-inferiority trial; or (2) equivalent to an existing treatment within a specified range, using an equivalence trial (Tables 5, 6 and Figure 1). Equivalence trails are not to be confused with bioequivalence trials where generic drug preparations are compared to currently marketed formulations with respect to their pharmacokinetic parameters.

**Table 5:** Information required to estimate a sample size for equivalence, superiority and non-inferiority trials.

**Outcome variable**	**Study design**	**Arguments**	**References**
Continuous	Equivalence trial	The expected mean of the outcome variable in the treatment and control groups, the expected population standard deviation of the outcome variable, the equivalence limit (expressed as a proportion), the required study power, the ratio of the number of exposed subjects to the number of unexposed subjects.	(15–17).
Binary	Equivalence trial	The expected proportion of successes in the treatment and control groups, the equivalence limit (expressed as a proportion), the required study power, the ratio of the number of exposed subjects to the number of unexposed subjects.	(15–17).
Continuous	Superiority trial	The expected mean of the outcome variable in the treatment and control groups, the expected population standard deviation of the outcome variable, the equivalence limit (expressed as a proportion), the required study power, the ratio of the number of exposed subjects to the number of unexposed subjects.	(15).
Binary	Superiority trial	The expected proportion of successes in the treatment and control groups, the equivalence limit (expressed as a proportion), the required study power, the ratio of the number of exposed subjects to the number of unexposed subjects.	(15)
Continuous	Non-inferiority trial	The expected mean of the outcome variable in the treatment and control groups, the expected population standard deviation of the outcome variable, the equivalence limit (expressed as a proportion), the required study power, the ratio of the number of exposed subjects to the number of unexposed subjects.	(15–17)
Binary	Non-inferiority trial	The expected proportion of successes in the treatment and control groups, the equivalence limit (expressed as a proportion), the required study power, the ratio of the number of exposed subjects to the number of unexposed subjects.	(16, 17)

**Table 6:** Formulae to estimate a sample size for equivalence, superiority and non-inferiority trials, binary or continuous population parameters.

**Outcome variable**	**Study design**	**Formula**	**Arguments**
Continuous	Equivalence trial	*n*_*A*_ = *rn*_*B*_ $n_{B}=\left(1+\;\frac{1}{r}\right)\;{\left(\sigma \frac{z_{1-\left(\alpha /2\right)}+\;z_{1-\beta /2}\;}{|\mu_{A}-\mu_{B}|-\delta }\right)}^{2}$ 1 − β = 2[Φ(*z* − *z*_1−(α/2)_) + Φ(−*z* − *z*_1−(α/2)_)]−1 $z=\;\frac{|\mu_{A}-\mu_{B}|-\delta }{\sigma \;\sqrt{\frac{1}{n_{A}}+\;\frac{1}{n_{B}}}}$	μ_*A*_ = the expected mean of the outcome in the treatment group. μ_*B*_ = the expected mean of the outcome in the control group. σ = the expected standard deviation of the outcome across treatment and control groups. *r* = anticipated number of subjects in the treatment group divided by the anticipated number of subjects in the control group. Φ = the standard Normal distribution function. Φ^−1^= the standard Normal quantile function. α = the Type I error, e.g. α = 0.05. β = the Type II error, e.g. β = 0.20. δ = the equivalence margin.
Binary	Equivalence trial	*n*_*A*_ = *rn*_*B*_ $n_{B}=\left(\frac{p_{A}\left(1-p_{A}\right)+\;p_{B}\left(1-p_{B}\right)\;}{r}\right)\;{\left(\sigma \frac{z_{1-\left(\alpha /2\right)}+\;z_{1-\beta /2}\;}{|p_{A}-p_{B}|-\delta }\right)}^{2}$ 1 − β = 2[ Φ(*z* − *z*_1 − (α/2)_) + Φ(−*z* − *z*_1−(α/2)_)]−1 $z=\;\frac{|p_{A}-p_{B}|-\delta }{\sigma \sqrt{\frac{p_{A}\left(1-p_{A}\right)}{n_{A}}+\;\frac{p_{B}\left(1-p_{B}\right)}{n_{B}}}}$	*p*_*A*_ = the expected probability of success in the treatment group. *p*_*B*_ = the expected probability of success in the control group. *r* = anticipated number of subjects in the treatment group divided by the anticipated number of subjects in the control group. Φ = the standard Normal distribution function. Φ^−1^= the standard Normal quantile function. α = the Type I error, e.g. α = 0.05. β = the Type II error, e.g. β = 0.20. δ = the equivalence margin.
Continuous	Superiority trial or non-inferiority trial	*n*_*A*_ = *rn*_*B*_ $n_{B}=\left(1+\;\frac{1}{r}\right)\;{\left(\sigma \frac{z_{1-\left(\alpha /2\right)}+\;z_{1-\beta }\;}{\;\mu_{A}-\mu_{B}-\delta }\right)}^{2}$ 1 − β = Φ(*z* − *z*_1−α_) + Φ(−*z* − *z*_1−α_) $z=\;\frac{\mu_{A}-\mu_{B}-\delta }{\sigma \;\sqrt{\frac{1}{n_{A}}+\;\frac{1}{n_{B}}}}$	μ_*A*_ = the expected mean of the outcome in the treatment group. μ_*B*_ = the expected mean of the outcome in the control group. σ = the expected standard deviation of the outcome across treatment and control groups. *r* = anticipated number of subjects in the treatment group divided by the anticipated number of subjects in the control group. Φ = the standard Normal distribution function. Φ^−1^= the standard Normal quantile function. α = the Type I error, e.g. α = 0.05. β = the Type II error, e.g. β = 0.20. δ = the equivalence margin.
Binary	Superiority trial or non-inferiority trial	*n*_*A*_ = *rn*_*B*_ $n_{B}=\left(\frac{p_{A}\left(1-p_{A}\right)+\;p_{B}\left(1-p_{B}\right)\;}{r}\right)\;{\left(\frac{z_{1-\left(\alpha /2\right)}+\;z_{1-\beta }\;}{p_{A}-p_{B}-\delta }\right)}^{2}$ 1 − β = Φ(*z* − *z*_1−(α/2)_) + Φ(−*z* − *z*_1−(α/2)_) $z=\;\frac{p_{A}-p_{B}-\delta }{\sqrt{\frac{p_{A}\left(1-p_{A}\right)}{n_{A}}+\;\frac{p_{B}\left(1-p_{B}\right)}{n_{B}}}}$	*p*_*A*_ = the expected probability of success in the treatment group. *p*_*B*_ = the expected probability of success in the control group. *r* = anticipated number of subjects in the treatment group divided by the anticipated number of subjects in the control group. Φ = the standard Normal distribution function. Φ^−1^= the standard Normal quantile function. α = the Type I error, e.g. α = 0.05. β = the Type II error, e.g. β = 0.20. δ = the equivalence margin. z_1−(α/2)_ = values from the standard normal curve corresponding to the desired level of confidence. Use z_1−(α/2)_ = 1.96 for 95% (two side) confidence. z_1−β_ = value from the standard normal curve corresponding to the desired study power. Use z_1−β_ = 0.84 for 80% power.

Superiority, non-inferiority and equivalence trials require the analyst to specify an equivalence margin. The equivalence margin is the range of values for which the treatment efficacies are close enough to be considered the same (19). Expressed in another way, the equivalence margin is the maximum clinically acceptable difference one is willing to accept in return for the secondary benefits of the new treatment.

Equivalence margins can be set on the basis of a clinical estimation of a minimally important effect. This approach is subjective and, as a result, it is possible to set the equivalence margin to be greater than the effect of the established treatment, which could lead to potentially harmful treatments classified as non-inferior. A second approach is to select an equivalence margin with reference to the effect of the established treatment in trials where a placebo has been used. When the equivalence margin is chosen in this way, there is some objective basis on which to claim that a positive non-inferiority trial implies that a new treatment is, in fact, superior to the established treatment (assuming the effect of the established treatment in the current trial is similar to its effect in the historical trials). An example sample size calculation for a non-inferiority trial is presented in Box 4.

Box 4 **Suppose a pharmaceutical company would like to conduct a clinical trial to compare the efficacy of two antimicrobial agents when administered orally to patients with skin infections. Assume the true mean cure rate of the treatment is 0.85 and the true mean cure rate of the control is 0.65. We consider a difference of <0.10 in cure rate to be of no clinical importance (i.e., delta = −0.10). Assuming a one-sided test size of 5% and a power of 80% how many subjects should be included in the trial?**

$n_{B}=\left(\frac{p_{A}\left(1-\;p_{A}\right)+\;p_{B}\left(1-\;p_{B}\right)\;}{r}\right)\;{\left(\frac{z_{1-\left(\alpha /2\right)}+\;z_{1-\;\beta }\;}{p_{A}-\;p_{B}-\;\delta }\right)}^{2}$

$\;n_{B}=\left(\frac{0.85\left(1-\;0.85\right)+\;0.65\left(1-\;0.65\right)\;}{1}\right)\;{\left(\frac{1.96+\;0.84\;}{0.85-\;0.65-\;-0.10}\right)}^{2}$

$\;n_{B}=\left(\frac{0.355\;}{1}\right)\;{\left(\frac{2.48\;}{0.30}\right)}^{2}$

       *n*_*B*_ = 25

A total of 50 subjects need to be enrolled in the trial, 25 in the treatment group and 25 in the control group.

### Sample Size Calculations to Detect the Presence of an Event

Sampling of individuals to either detect the presence of an event (usually the presence of disease or the presence of infection) or provide evidence that disease is absent from a jurisdiction are frequent activities in veterinary epidemiology. Typical scenarios include: (1) shipment of live animals from one country to another where the country receiving the shipment might request that testing is carried out on a sample of individuals, as opposed to testing every animal; and (2) a country wishing to re-gain official disease freedom status following an infectious disease outbreak.

Details of formula-based sample size estimation methods to detect the presence of an event are provided in Table 7. Sample size estimation methods can be categorized into two groups: (1) to ensure sufficient units are sampled to return a desired (posterior) probability of disease freedom; and (2) to ensure sufficient units are sampled to ensure a surveillance system has a desired system sensitivity. For the surveillance system sensitivity methods sampling can be either representative or risk based. All of the methods listed in Table 7 account for imperfect diagnostic test sensitivity at the surveillance unit level.

**Table 7:** Summary of sample size formulae to estimate the probability of disease freedom or estimate surveillance system sensitivity.

**Study design**	**Outcome**	**Arguments**	**References**
Representative sampling	Probability of disease freedom assuming imperfect test sensitivity and perfect test specificity.	The assumed population size, an estimate of the prior probability that the population is free of disease, an estimate of the probability of disease introduction, the population level design prevalence, the desired probability that the population is free of disease, the sensitivity of the diagnostic test used at the surveillance unit level.	(22, 23)
Representative sampling	Surveillance system sensitivity assuming a single risk factor and varying test sensitivity.	The assumed population size, the population level design prevalence, the desired surveillance system sensitivity, the sensitivity of the diagnostic test used at the surveillance unit level.	(24, 25)
Representative sampling	Surveillance system sensitivity assuming two stage sampling, imperfect test sensitivity and perfect test specificity.	The number of clusters in the population, the number of surveillance units within each cluster, the cluster level design prevalence, the desired cluster level sensitivity, the population level design prevalence, the desired population level sensitivity, the sensitivity of the diagnostic test used at the surveillance unit level.	(24–27)
Representative sampling	Surveillance system sensitivity, imperfect test sensitivity and imperfect test specificity.	The assumed population size, the population level design prevalence, the desired population level sensitivity, the desired population level specificity, the sensitivity of the diagnostic test at the surveillance unit level, the specificity of the diagnostic test at the surveillance unit level.	(26–28)
Representative sampling	Surveillance system sensitivity assuming pooled sampling giving rise to imperfect test sensitivity and imperfect test specificity.	The number of surveillance units that contribute to each pool, the population level design prevalence, the sensitivity of the diagnostic test at the pooled level, the specificity of the diagnostic test at the pooled level, the desired population level sensitivity.	(29)
Risk-based sampling	Surveillance system sensitivity assuming imperfect test sensitivity and perfect test specificity.	The population level design prevalence, relative risk estimates for each strata, the population proportions for each strata, the surveillance proportions for each strata, the desired population level sensitivity, the sensitivity of the diagnostic test at the surveillance unit level.	(5)
Risk-based sampling	Surveillance system sensitivity assuming risk-based 2-stage sampling on one risk factor at the cluster level assuming imperfect test sensitivity and perfect test specificity.	Relative risk values for each strata in the population, the population proportions in each strata, the planned number of units to be sampled from each strata, the cluster level design prevalence, the desired cluster level sensitivity, the surveillance unit level design prevalence, the sensitivity of the diagnostic test at the surveillance unit level, the desired surveillance system (population-level) sensitivity.	(5)
Risk-based sampling	Surveillance system sensitivity assuming risk-based 2-stage sampling on two risk factors at either the cluster level, the unit level or both, imperfect test sensitivity and perfect test specificity.	The number of risk strata defining the relative risk values at the cluster level, the population proportions at the cluster level, the planned surveillance proportions at the cluster level, the cluster level design prevalence, the desired cluster level sensitivity, the number of risk strata defining the relative risk values at the surveillance unit level, the population proportions at the surveillance unit level, the planned surveillance proportions at the surveillance unit level, the surveillance unit level design prevalence, the sensitivity of the diagnostic test at the surveillance unit level, the desired surveillance system (population-level) sensitivity.	(5)

When tests with both imperfect diagnostic sensitivity and specificity are used, diseased individuals can be missed because of imperfect diagnostic sensitivity but at the same time disease negative individuals can be incorrectly identified as disease positive because of imperfect specificity. Cameron and Baldock (26) describe an approach to estimate the number of animals to be sampled from a finite population using a test with imperfect diagnostic sensitivity and specificity using the hypergeometric distribution. This method returns the number of individuals to be sampled and the estimated probability that the population is diseased for 1 to *n* individuals that return a positive test result. This allows an analyst to make a statement that they can be (for example) 95% confident that the prevalence of disease in the population of interest is less than the stated design prevalence if the number of (surveillance) units with a positive test result is less than or equal to a specified cut-point. A worked example of this approach is provided in the Supplementary Material.

## Sample Size Calculations for Clustered Data

Aggregation of individual sampling units into groups (“clusters”) for example farms, households or villages violates the assumption of independence that is central to the sample size calculation methods described so far. When individuals are aggregated into clusters there are two sources of variation in the outcome of interest. The first arises from the effect of the cluster; the second from the effect of the individual. This means that individuals selected from the same cluster are more likely to be similar compared with those sampled from the general population (30). For this reason, the effective sample size when observations are made on randomly selected individuals from the same cluster will be less than that when observations are made on individuals selected completely at random from the general population. For studies where the objective is to estimate a population parameter (e.g., a prevalence) a reduction in effective sample size increases the uncertainty around the estimate of the population parameter. For studies where the objective is to test a hypothesis a reduction in effective sample size results in a reduction in statistical power, in effect the ability to detect a statistically significant difference in event outcomes for exposure positive and exposure negative individuals given a true difference actually exists.

With one-stage cluster sampling a random sample of clusters is selected first and then all individual listing units within each cluster are selected for study. With two-stage cluster sampling a random sample of clusters is selected first and then a random sample of individual listing units within each cluster is selected. The primary advantage of cluster sampling is logistics. In animal health, where animals are typically managed within clusters (e.g., herds or flocks) it is easier to select clusters first and then, from each selected cluster, take a sample of individual animals. This contrasts with a simple random sampling approach which would require an investigator to travel to a large number of herds-flocks, sampling small numbers of animals from each. As explained above, the main disadvantage of cluster sampling is a reduction in the effective sample size due to animals from the same cluster being more homogenous (similar) compared with those from different clusters.

To compensate for this lack of precision Donner et al. (31) proposed that a sample size estimate calculated assuming complete independence (using the formulae presented in Tables 1–7) can be inflated by a value known as the design effect (*D*) to achieve the level of statistical power achieved using independent sampling. For a single level of clustering (e.g., the situation where cows are clustered within herds) the design effect is calculated as:

(1) $$\begin{array}{r} D=1+\left(b-1\right)\rho \end{array}$$

In Equation 1 *b* equals the number of animals to be sampled from each cluster (not to be confused with the total number of animals eligible for sampling within each cluster) and ρ is the intracluster correlation coefficient (ICC). The value of ρ equals the between-cluster variance $\sigma_{B}^{2}$ divided by the between-cluster variance plus the within-cluster variance ($\left(\sigma_{B}^{2}+\;\sigma_{W}^{2}\right)$ :

(2) $$\begin{array}{r} \rho =\;\frac{\sigma_{B}^{2}}{\sigma_{B}^{2}+\;\sigma_{W}^{2}} \end{array}$$

When there is little variation in an outcome within a cluster (e.g., observations made on individual cows within herds are “similar,” $\sigma_{W}^{2}$ will be small) ρ will be close to 1 and the design effect will therefore be large. When there is wide variation within a cluster (e.g., observations made on individual cows within herds showing a similar variability to the general population, $\sigma_{W}^{2}$ will be large) ρ will be close to 0 and, therefore, the design effect will be close to unity. Using the definition provided above (Equation 2), ρ ranges between 0 and +1 with typical values ranging from 0 to 0.05 for non-communicable diseases and values >0.4 uncommon. Papers providing ICC estimates for various outcomes in the human and veterinary literature have been published: see, for example, (20, 32, 33). Researchers should be aware of the importance of publishing estimates of ICC since high quality empirical data are necessary to provide credible sample size estimates for future studies. More importantly, for the same outcome measure, ICC estimates will vary from one research setting to another so access to a likely range of ICC measures is desirable.

A number of methods are available to estimate ρ from empirical data (34, 35) ranging from one-way analysis of variance (36) to regression-based approaches using mixed effects models (37). Eldridge and Kerry (38) provide a comprehensive review of appropriate techniques.

An example of how the ICC can be used to estimate the number of primary sampling units for a one-stage cluster design is provided in Box 5.

Box 5 **An aid project has distributed cook stoves in a single province in a resource-poor country. At the end of 3 years, the donors would like to know what proportion of households are still using their donated stove. A cross-sectional study is planned where villages in a province will be sampled and all households (~75 per village) will be visited to determine if the donated stove is still in use. A pilot study of the prevalence of stove usage in five villages showed that 0.46 of householders were still using their stove and the ICC for stove use within villages is in the order of 0.20. If the donor wanted to be 95% confident that the survey estimate of stove usage was within 10% of the true population value, how many villages (clusters) need to be sampled?**

*D* = 1 + (*b* − 1)ρ

*D* = 1 + (75 − 1) × 0.20

*D* = 15.8

$n_{c}\geq \frac{{z_{1-\left(\alpha /2\right)}}^{2}\;P_{y}\;\left(1-\;P_{y}\right)D}{{\left(P_{y}\;\epsilon_{r}\right)}^{2}b}$

$n_{c}\geq \frac{1.96^{2}\;0.46\;\left(1-\;0.46\right)15.8}{{\left(0.46\;\times 0.10\right)}^{2}\;\times \;75}$

$n_{c}\geq \frac{15.077}{0.1587}$

*n*_*c*_ ≥ 96

A total of 96 villages need to be sampled to meet the requirements of the study.

The example shown in Box 5 is somewhat unrealistic in that it is assumed that the number of households in each village is a constant value of 75. Eldridge, Ashby and Kerry (39) provide an approach to estimate a sample size using a one-stage cluster design when the number of individual listing units per cluster varies (Box 6).

Box 6 **Continuing the example presented in Box 5, we are now told that the number of households per village varies. The average number of households per village is 75 with a 0.025 quartile of 40 households and a 0.975 quartile of 180. Assuming the number of households per village follows a normal distribution the expected standard deviation of the number of households per village is in the order of (180 – 40) ÷ 4 = 35. How many villages need to be sampled? In the formula below, CV standards for coefficient of variation defined as the standard deviation of the cluster sizes divided by the mean of the cluster sizes.**

$D=1+\left\{\left(CV^{2}+1\right)\;b\;-1\right\}\rho$

    *D* = 1 + {(0.467^2^ + 1) 75 − 1}0.2

    *D* = 19.1

    $n_{c}\geq \frac{{z_{1-\left(\alpha /2\right)}}^{2}\;P_{y}\;\left(1-\;P_{y}\right)D}{{\left(P_{y}\;\epsilon_{r}\right)}^{2}b\;}$

    $n_{c}\geq \frac{1.96^{2}\;0.46\;\left(1-\;0.46\right)19.1}{{\left(0.46\;\times 0.10\right)}^{2}\;\times \;75}$

    $n_{c}\geq \frac{18.194}{0.1587}$

    *n*_*c*_ ≥ 115

A total of 115 villages need to be sampled to meet the requirements of the study.

An example showing how a crude sample size estimate (i.e., a sample size calculated assuming independence) can be adjusted to account for clustering using the design effect is provided in Box 7.

Box 7 **Continuing the example provided in Box 1, being seropositive to brucellosis is likely to cluster within herds. Otte and Gumm (20) cite the intracluster correlation coefficient for *Brucella abortus* in cattle to be in the order of 0.09. We now adjust our sample size estimate of 545 to account for clustering at the herd level. Assume that, on average, *b* = 20 animals will be sampled per herd:**

*D* = 1 + (*b* − 1)ρ

*D* = 1 + (20 − 1) × 0.09

*D* = 2.71

After accounting for the presence of clustering at the herd level we estimate that a total of (545 × 2.71) = 1,477 cattle need to be sampled to meet the requirements of the survey. If 20 cows are sampled per herd this means that a total of (1,477 ÷ 20) = 74 herds are required.

Three levels of clustering are relatively common in veterinary epidemiological research (much more so than in human epidemiology) where, for example, lactations (level 1 units) might be sampled within cows (level 2 units) which are then sampled within herds (level 3 units). The total variance in this situation is made up of the variance associated with lactations within cows within herds $\sigma_{1}^{2}$, the variance between cows within herds $\sigma_{2}^{2}$, and the variance between herds $\sigma_{3}^{2}$. Two ICCs can be calculated: lactations within herds:

(3) $$\begin{array}{r} \rho_{2}=\;\frac{\sigma_{3}^{2}}{\sigma_{3}^{2}+\;\sigma_{2}^{2}+\;\sigma_{1}^{2}} \end{array}$$

and lactations within cows:

(4) $$\begin{array}{r} \rho_{1}=\;\frac{\sigma_{3}^{2}+\;\sigma_{2}^{2}}{\sigma_{3}^{2}+\;\sigma_{2}^{2}+\;\sigma_{1}^{2}} \end{array}$$

In a study comprised of three levels the required sample size, accounting for clustering equals (40):

(5) $$\begin{array}{r} n_{3}n_{2}n_{1}=\;DE\;\times m \end{array}$$

In Equation 5, *m* is the number of lactations to be sampled to meet the requirements of the study assuming the data are completely independent and *n*_3_, *n*_2_, and *n*_1_ are the number of units to be sampled at the herd, cow and lactation level (respectively). The design effect for three levels of clustering equals:

(6) $$\begin{array}{r} DE=1+n_{1}\left(n_{2}-1\right)\rho_{2}+\;\left(n_{1}-1\right)\rho_{1} \end{array}$$

Box 8 provides a worked example of a sample size calculation for the three-level clustering scenario.

Box 8 **Dohoo et al. (21) provide details of an observational study of the reproductive performance of dairy cows on Reunion Island. If this study were to be repeated, how many lactations would need to be sampled to be 95% confident that the estimated logarithm of calving to conception interval was within 5% of the true population value?**

From (21) the standard deviations of the random effect terms from a multilevel model of factors influencing log transformed calving to conception interval at the herd, cow and lactation level were 0.1157, 0.1479, and 0.5116, respectively. The ICC for lactations within herds (Equation 3):

$$\begin{array}{r} \rho_{2}=\;\frac{\sigma_{3}^{2}}{\sigma_{3}^{2}+\;\sigma_{2}^{2}+\;\sigma_{1}^{2}}\\ \rho_{2}=\;\frac{0.1157^{2}}{0.1157^{2}+\;0.1479^{2}+\;0.5116^{2}}\\ \rho_{2}=\;0.0451 \end{array}$$

and the ICC for lactations within cows (Equation 4):

$$\begin{array}{r} \rho_{1}=\;\frac{\sigma_{3}^{2}+\;\sigma_{2}^{2}}{\sigma_{3}^{2}+\;\sigma_{2}^{2}+\;\sigma_{1}^{2}}\\ \rho_{1}=\;\frac{0.1157^{2}+\;0.1479^{2}}{0.1157^{2}+\;0.1479^{2}+\;0.5116^{2}}\\ \rho_{1}=\;0.1188 \end{array}$$

The mean and standard deviation of the logarithm of calving to conception interval was 4.59 and 0.54, respectively. What is the required sample size assuming the data are independent?

$$\begin{array}{l} m_{1}\geq \frac{1.96\times 1,000,000\;\times (0.54^{2}÷4.59^{2})\;}{1.96\;(0.54^{2}÷4.59^{2})+(\left[1,000,000-1\right]\;0.05^{2})}\\ m_{1}\geq \frac{54057.4}{2500.025}\\ m_{1}\geq 22 \end{array}$$

Assuming the data are independent a total of 22 lactations are required to be 95% confident that our estimate of the logarithm of calving to conception interval is within 5% of the true population value.

We elect to sample two lactations per cow. How many lactations are required to account for clustering of lactations within cows?

$$\begin{array}{r} n_{1}=\;2\\ D_{1}=\;1+\;\rho_{1}\left(n_{1}-1\right)\\ D_{1}=1+\;0.1188\left(2-1\right)\\ D_{1}=1.1188\\ m_{2}=D_{1}\;\times \;m_{1}\\ m_{2}=1.1188\;\times 22\\ m_{2}=25 \end{array}$$

A total of 25 lactations are required accounting for clustering of lactations within cows. How many cows are required?

$$\begin{array}{r} n_{2}=\;m_{2}\;/\;n_{1}\\ n_{2}=\;25\;/\;2\\ n_{2}=\;13 \end{array}$$

A total of 13 cows are required if we sample two lactations per cow (26 lactations in total).

We now consider clustering at the herd level. How many lactations are required to account for clustering of cows within herds?

$$\begin{array}{r} D_{2}=\;1+\;\left(n_{1\;}\times \left(n_{2}-1\right)\times \;\rho_{2}\right)+\left(\left(n_{1}-1\right)\;\times \;\rho_{1}\right)\\ D_{2}=\;1+\;\left(2\times \left(13-1\right)\times \;0.0451\right)+\left(\left(2-1\right)\;\times \;0.1188\right)\\ D_{2}=\;2.2016\\ m_{3}=D_{2}\;\times \;m_{1}\\ m_{3}=2.2061\;\times \;22\\ m_{3}=49 \end{array}$$

Accounting for clustering of lactations within cow and cows within herds, a total of 39 lactations are required. How many herds are required?

$$\begin{array}{r} n_{3}=\;m_{3}\;/\;\left(n_{1}\;\times \;n_{2}\right)\\ n_{3}=\;49\;/\;\left(2\;\times 13\right)\\ n_{3}=\;2 \end{array}$$

A total of 2 herds are required if we sample 13 cows from each herd and 2 lactations from each cow. The total number of lactations required is therefore:

$$\begin{array}{r} n_{total}=\;\left(n_{1}\;\times \;n_{2}\;\times \;n_{3}\right)\\ n_{total}=\;\left(2\;\times \;13\;\times \;2\right)\\ n_{total}=\;52 \end{array}$$

To account for lack of independence in the data arising from clustering of lactations within cows and cows within herds 52 lactations (2 lactations from 13 cows from 2 herds) are required to meet the requirements of the study.

The required sample size assuming the data were independent was 22. The required sample size accounting for lack of independence in the data was 52, a 2.5-fold difference.

## Simulation-Based Approaches for Sample Size Estimation

In applied veterinary epidemiological research it is common for study designs not to conform to the standard study designs for which sample size formulae are available. Typical examples include situations where study subjects are organized into more than three levels of aggregation and in clinical trials where a treatment might be applied at the group level and a second treatment applied at the individual level. Where there are multiple levels of aggregation researchers may elect to apply a more conservative design effect multiplier than that used when study subjects are kept in simpler cluster groups. While this approach is an attempt to address the problem, it can result in the final sample size estimate being larger than the final sample size required if the design effect was known more precisely.

For complex study designs simulation-based approaches provide an alternative for sample size estimation that is relatively easy to implement using modern statistical software (41). In the text that follows a worked example is provided, where simulation is used to estimate the number of lactations, cows and herds to be sampled to provide an estimate of log calving to conception interval, using the scenario presented in Box 8.

The general approach when using simulation to estimate a sample size to estimate a population parameter is to: (1) simulate a population data set that respects clustering of the outcome variable within the population of interest; (2) define a series of candidate sample size estimates; (3) repeatedly sample the simulated population using each of the candidate sample size estimates to determine the proportion of occasions the estimate of the population parameter is within the prescribed relative error of the true population value. When estimating a population prevalence and assuming the level of confidence specified by the analyst has been set to 95%, the required sample size is the combination of level 1, 2, 3, . *n* units sampled that returns an estimate of the outcome variable that is within the prescribed relative error of the true population value on 95% of occasions. Note that several different combinations of units sampled at each level might achieve the stated objectives of the study.

When the study aim is to test a hypothesis, an additional step is to assign the exposure variable (e.g., a treatment) to members of the population and then to estimate the effect of the exposure on the outcome of interest using a regression approach. Arnold et al. (41) provide a worked example of this approach using a two-treatment factorial trial in rural Bangladesh as an example. In this study children <6 months of age were randomly assigned to one of four treatment groups: control, sanitation mobilization, lipid-based nutrient supplementation, and sanitation plus lip-based nutrient supplementation. The design of this study made sample size and study power calculations difficult for two reasons: (1) treatments were deployed at two levels (sanitation mobilization at the community level and lipid supplementation at the individual level); and (2) there were two sources of correlation in the outcome: at the community level and the individual child level.

Generation of the population data set involves: (1) defining the mean and standard deviation of the outcome of interest (for continuous outcomes) or the expected population prevalence (for binary outcomes); (2) defining the number of level 1, 2, 3, ... *n* units in the population; (3) defining the level 1, 2, 3, ... *n* variance terms; (4) simulating a population of individuals eligible for sampling based on the specified number of level 1, 2, 3, ... *n* units; (5) assignment of a value for the outcome variable to each member of the simulated population, and; (6) adjustment of the value of the outcome variable for each individual to account for clustering using the level 1, 2, 3, ... *n* variance terms.

Code written in the R programming language (6) to generate a population data set for the Reunion Island dairy cow reproduction example (25) and estimate a sample size to meet the requirements of the study is provided in the Supplementary material accompanying this paper.

**Figure 2:**
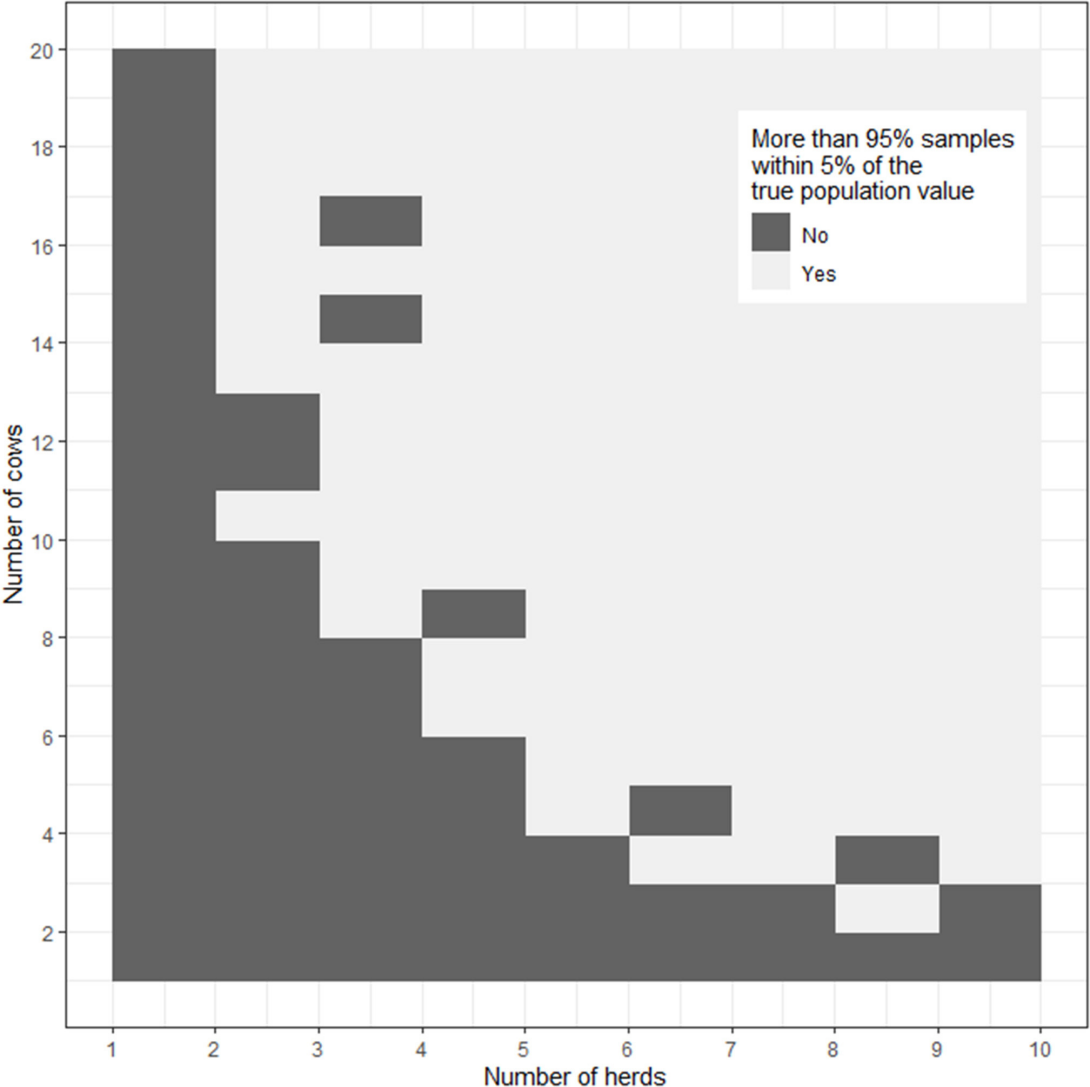
Image plot showing the percentage of simulations where the sample estimate of the logarithm of calving to conception interval was within 5% of the true population value as a function of the number of herds and number of cows from each herd sampled. The dashed line shows the herd-cow sample size combinations where >95% of simulations returned an estimate of the logarithm of calving to conception interval that was within 5% of the true population value.

Figure 2 is an image plot showing the proportion of simulations where the sample estimate of the logarithm of calving to conception interval was within 5% of the true population value as a function of the number of sampled herds and the number of cows sampled from within each herd. In Figure 2 the superimposed contour line shows the herd-cow sample size combinations where >95% of simulations returned an estimate of the logarithm of calving to conception interval that was within 5% of the true population value, in agreement with the requirement for 2 lactations from 13 cows from 2 herds (*n* = 52 lactations) calculated using the formula-based approach presented in Box 8. When the results of simulations are presented in this way one can appreciate that there is some flexibility in the combinations of herd and cow numbers that need to be sampled to meet the requirements of the study. For example, Figure 2 shows that the estimate of mean logarithm of calving to conception interval would be within 5% of the true population value if a smaller number of cows (e.g., *n* = 6) were sampled from a larger number of herds (e.g., *n* = 6).

In summary, the process of simulation replaces the time and effort to derive a formula-based approach for a complex study design with basic programming and computer simulation time. An additional positive side effect is that the process of simulation requires investigators to define the structure of their study population, the expected value and variability of the outcome of interest and how the results of the study will be analyzed once the data are collected. This reduces the likelihood of investigators exploring alternative analytical approaches in the presence of negative findings, consistent with CONSORT guidelines (42).

## Conclusions

This paper has provided an overview of the reasons researchers might need to calculate an appropriate sample size in veterinary epidemiology and a summary of different sample size calculation methods. In contrast to human epidemiology individual study subjects in veterinary epidemiology are almost always aggregated into hierarchical groups (43) and, for this reason, sample size estimates calculated using simple formulae that assume independence are usually not appropriate in a veterinary setting. This paper provides details of two approaches for dealing with this problem: (1) inflation of a crude sample size estimate using a design effect; and (2) use of a simulation-based approaches. The key advantage of simulation-based approaches is that appropriate sample sizes can be estimated for complex study designs for which formula-based methods are not available.

## Author Contributions

The author confirms being the sole contributor of this work and has approved it for publication.

## Conflict of Interest

The author declares that the research was conducted in the absence of any commercial or financial relationships that could be construed as a potential conflict of interest.
