# Supplementary material for: Sample Size Estimation in Veterinary Epidemiologic Research
Source: Front Vet Sci. 2021 Feb 17;7:539573. doi: 10.3389/fvets.2020.539573 (PMC7925405; doi:10.3389/fvets.2020.539573)
Supplement: Supplementary file 1 [file Data_Sheet_1.pdf]

Sample size estimation in veterinary epidemiologic research

Mark Stevenson

2020-12-19

The following code requires `epiR` (version 2.0.17 or higher). To download the latest version go to https://cran.r-project.org/web/packages/epiR/index.html.

Box 1: Prevalence estimation

The expected seroprevalence of brucellosis in a population of cattle is thought to be in the order of 15%. How many cattle need to be sampled and tested to be 95% certain that our seroprevalence estimate is within 20% (i.e. 0.20 $\times$ 0.15 = 0.03, 3%) of the true population value, assuming use of a test with perfect sensitivity and specificity? This formula requires the population size to be specified so we set N to a large number, 1,000,000:

```
library(epiR); library(ggplot2); library(nlme); library(RColorBrewer); library(readstata13)

z <- 1.96
N <- 1000000
Py <- 0.15
epsilon.r <- 0.2

num <- z^2 * N * Py * (1 - Py)
den <- ((N - 1) * epsilon.r^2 * Py^2) + (z^2 * Py * (1 - Py))
n <- ceiling(num / den); n
#> [1] 544

epi.sssimpleestb(N = 1E+06, Py = 0.15, epsilon.r = 0.20, se = 1, sp = 1, nfractional =
         FALSE, conf.level = 0.95)
#> [1] 545
```

Minor differences between the sample size estimates calculated manually and those from `epiR` due to rounding.

Box 2: Prospective cohort study

A prospective cohort study of dry food diets and feline lower urinary tract disease (FLUTD) in mature male cats is planned. A sample of cats will be selected at random from the population and owners who agree to participate in the study will be asked to complete a questionnaire at the time of enrolment. Cats enrolled into the study will be followed for at least 5 years to identify incident cases of FLUTD. The investigators would like to be 0.80 certain of being able to detect when the risk ratio of FLUTD is 1.4 for cats habitually fed a dry food diet, using a 0.05 significance test. Previous evidence suggests that the incidence risk of FLUTD in cats not on a dry food (i.e. ‘other’) diet is around 50 per 1000 per year. Assuming equal numbers of cats on dry food and other diets are sampled, how many cats should be enrolled into the study?

```
irexp0 <- 50 / 1000
irexp1 <- 70 / 1000
FT <- 5
r <- 1

z.beta <- 0.84
z.alpha <- 1.96

lambda0 <- irexp0
lambda1 <- irexp1
lambda <- mean(c(lambda1, lambda0))

flambda0 <- (lambda0^3 * FT) / ((lambda0 * FT) - 1 + exp(-lambda0 * FT))
flambda1 <- (lambda1^3 * FT) / ((lambda1 * FT) - 1 + exp(-lambda1 * FT))
flambda <- (lambda^3 * FT) / ((lambda * FT) - 1 + exp(-lambda * FT))

num <- (z.alpha * sqrt((1 + r) * flambda) + z.beta * sqrt((r * flambda1 + flambda0)))^2
den <- (r * (lambda1 - lambda0)^2)
n <- ceiling(num / den); n
#> [1] 1039

epi.sscohortt(irexp1 = 50/1000, irexp0 = 70/1000, FT = 5, n = NA, power = 0.80, r = 1,
   design = 1, sided.test = 2, nfractional = FALSE, conf.level = 0.95)$n.total
#> [1] 2080
```

A total of 2080 male cats need to be sampled to meet the requirements of the study (1040 cats habitually fed dry food and 1040 cats habitually fed ‘other’ diet types).

Box 3: Case-control study

A case-control study of the association between white pigmentation around the eyes and ocular squamous cell carcinoma in Hereford cattle is planned. A sample of cattle with newly diagnosed squamous cell carcinoma will be compared for white pigmentation around the eyes with a sample of controls. Assuming an equal number of cases and controls, how many study subjects are required to detect an odds ratio of 2.0 with 0.80 power using a two-sided 0.05 test? Previous surveys have shown that around 0.30 of Hereford cattle without squamous cell carcinoma have white pigmentation around the eyes.

```
z.beta  <- 0.84
z.alpha <- 1.96
OR <- 2
r <- 1
p0 <- 0.3

Pc <- (p0 / (r + 1)) * ((r * OR / (1 + (OR - 1) * p0)) + 1)
t1 <- (r + 1) * (1 + (OR - 1) * p0)^2
t2 <- r * p0^2 * (p0 - 1)^2 * (OR - 1)^2
t3 <- z.alpha * sqrt((r + 1) * Pc * (1 - Pc))
t4 <- OR * p0 * (1 - p0)
t5 <- 1 + (OR - 1) * p0
t6 <- t4 / (t5^2)
t7 <- r * p0 * (1 - p0)
```

```r
t8 <- z.beta * sqrt(t6 + t7)
n <- ceiling((t1 / t2) * (t3 + t8)^2); n
#> [1] 281

epi.sscc(OR = 2.0, p0 = 0.30, n = NA, power = 0.80,
   r = 1, rho = 0, design = 1, sided.test = 2, conf.level = 0.95,
   method = "unmatched", nfractional = FALSE, fleiss = FALSE)$n.total
#> [1] 282
```

If the true odds for squamous cell carcinoma in exposed subjects relative to unexposed subjects is 2.0, we will need to enrol 141 cases and 141 controls (282 cattle in total) to reject the null hypothesis that the odds ratio equals one with probability (power) 0.80. The Type I error probability associated with this test of this null hypothesis is 0.05.

Box 4: Non-inferiority trial

Suppose a pharmaceutical company would like to conduct a clinical trial to compare the efficacy of two antimicrobial agents when administered orally to patients with skin infections. Assume the true mean cure rate of the treatment is 0.85 and the true mean cure rate of the control is 0.65. We consider a difference of less than 0.10 in cure rate to be of no clinical importance (i.e. delta = -0.10). Assuming a one-sided test size of 5% and a power of 80% how many subjects should be included in the trial?

```r
treat = 0.85
control = 0.65
z.alpha <- 1.64
z.beta <- 0.84
delta <- -0.10

t1 <- treat * (1 - treat) +  control * (1 - control)
t2 <- r
t3 <- z.alpha + z.beta
t4 <- treat - control - delta
n <- ceiling((t1 / t2) * (t3 / t4)^2); n
#> [1] 25

epi.ssninfb(treat = 0.85, control = 0.65, delta = -0.10, n = NA,
   r = 1, power = 0.80, nfractional = FALSE, alpha = 0.05)$n.total
#> [1] 50
```

A total of 50 subjects need to be enrolled in the trial, 25 in the treatment group and 25 in the control group.

Freecalc

A cross-sectional study is to be carried out to confirm the absence of brucellosis in dairy herds using a bulk milk tank test assuming a design prevalence of 5%. Assume the total number of dairy herds in your study area is unknown and large and the bulk milk tank test to be used has a diagnostic sensitivity of 0.95 and a specificity of 1.00. How many herds need to be sampled if you want to be 95% certain that the prevalence of brucellosis in dairy herds is less than the design prevalence if all tests are negative?

```r
rsu.sssep.rs(N = NA, pstar = 0.05, se.p = 0.95, se.u = 0.95)
#> [1] 62
```

A total of 62 herds need to be sampled and tested.

Imagine the test we're using has a diagnostic sensitivity of 0.95 (as before) but this time it has a specificity of 0.98. How many herds need to be sampled to be 95% certain that the prevalence of brucellosis in dairy herds is less than the design prevalence if less than a specified number of tests return a positive result?

```r
rsu.sssep.rsfreecalc(N = 5000, pstar = 0.05, mse.p = 0.95,
   msp.p = 0.95, se.u = 0.95, sp.u = 0.98, method = "hypergeometric",
   max.ss = 32000)$summary
#>     n    N c pstar         p1     se.p      sp.p
#> 1 194 5000 7  0.05 0.04898102 0.951019 0.9573939
```

A system sensitivity of 95% is achieved with a total sample size of 194 herds, assuming a cut-point of 7 or more positive herds are required to return a positive survey result.

Note the substantial increase in sample size when diagnostic specificity is imperfect (194 herds when specificity is 0.98 compared with 63 when specificity is 1.00). The relatively low design prevalence in combination with imperfect imperfect specificity means that false positives are more likely to be a problem in this population so the number tested needs to be (substantially) increased.

Box 5: One-stage cluster sampling

An aid project has distributed cook stoves in a single province in a resource-poor country. At the end of three years, the donors would like to know what proportion of households are still using their donated stove. A cross-sectional study is planned where villages in a province will be sampled and all households (approximately 75 per village) will be visited to determine if the donated stove is still in use. A pilot study of the prevalence of stove usage in five villages showed that 0.46 of householders were still using their stove and the intracluster correlation coefficient (ICC) for stove use within villages is in the order of 0.20. If the donor wanted to be 95% confident that the survey estimate of stove usage was within 10% of the true population value, how many villages (clusters) need to be sampled?

```r
b <- 75
Py <- 0.46
epsilon.r <- 0.10
rho <- 0.20
z <- 1.96

D <- 1 + ((b - 1) * rho)
n.psu <- ceiling((z^2 * Py * (1 - Py)) * D / ((Py * epsilon.r)^2 * b)); n.psu
#> [1] 96

epi.ssclus1estb(b = 75, Py = 0.46, epsilon.r = 0.10, rho = 0.20, conf.level = 0.95)$n.psu
#> [1] 96
```

A total of 96 villages need to be sampled to meet the requirements of the study.

Box 6: One-stage cluster sampling (continued)

Continuing the example presented in Box 5, we are now told that the number of households per village varies. The average number of households per village is 75 with a 0.025 quartile of 40 households and a 0.975 quartile of 180. Assuming the number of households per village follows a normal distribution the expected standard deviation of the number of households per village is in the order of (180 - 40) ÷ 4 = 35. How many villages need to be sampled? In the code below, `bcv` standards for coefficient of variation defined as the standard deviation of the cluster sizes divided by the mean of the cluster sizes.

```
bmu <- 75
bsigma <- 35
Py = 0.46
epsilon.r <- 0.10
rho <- 0.20
z <- 1.96

bcv <- bsigma / bmu
D <- 1 + ((bcv^2 + 1) * bmu - 1) * rho
n.psu <- ceiling((z^2 * Py * (1 - Py)) * D / ((Py * epsilon.r)^2 * bmu)); n.psu
#> [1] 115

epi.ssclus1estb(b = c(75,35), Py = 0.46, epsilon.r = 0.10, rho = 0.20, conf.level =
         0.95)$n.psu
#> [1] 115
```

A total of 115 villages need to be sampled to meet the requirements of the study.

Box 7: Design effect

Continuing the example provided in Box 1, being seropositive to brucellosis is likely to cluster within herds. Otte and Gumm (1997) cite the intracluster correlation coefficient for *Brucella abortus* in cattle to be in the order of 0.09. We now adjust our sample size estimate of 545 to account for clustering at the herd level. Assume that, on average, b = 20 animals will be sampled per herd:

```
z <- 1.96
N <- 1000000
Py <- 0.15
epsilon.r <- 0.2
D <- 1 + ((20 - 1) * 0.09)

num <- z^2 * N * Py * (1 - Py)
den <- ((N - 1) * epsilon.r^2 * Py^2) + (z^2 * Py * (1 - Py))
n <- ceiling((num / den) * D); n
#> [1] 1475

ceiling(epi.sssimpleestb(N = 1E+06, Py = 0.15, epsilon.r = 0.20, se = 1, sp = 1,
   nfractional = FALSE, conf.level = 0.95) * D)
#> [1] 1477
```

After accounting for the presence of clustering at the herd level we estimate that a total of (545 × 2.71) = 1477 cattle need to be sampled to meet the requirements of the survey. If 20 cows are sampled per herd this means that a total of (1477 ÷ 20) = 74 herds are required.

Box 8: Hierarchical data

Dohoo et al. (2001) provide details of an observational study of the reproductive performance of dairy cows on Reunion Island. If this study were to be repeated, how many lactations would need to be sampled to be 95% confident that the estimated logarithm of calving to conception interval was within 5% of the true population value?

From Dohoo et al. (2001) the standard deviations of the random effect terms from a multilevel model of factors influencing log transformed calving to conception interval at the herd, cow and lactation level were 0.1157, 0.1479 and 0.5116, respectively. A copy of the Reunion Island dairy cow fertility data set (`reu_cc.dta`) is available from https://projects.upei.ca/ver/. This next code chunk can be skipped if you don't have access to the data:

```
dat <- read.dta13("reu_cc.dta", convert.factors = FALSE)

# Number of herds:
q3 <- length(unique(dat$herd)); q3
#> [1] 50

# Number of cows:
q2 <- length(unique(dat$cow));  q2
#> [1] 1345

# Number of lactations:
q1 <- length(unique(dat$obs));  q1
#> [1] 2509

# Frequency histogram of calving to conception intervals:
par(mfrow = c(1,2), pty = "s")
hist(exp(dat$lncc), xlab = "CC interval", ylim = c(0,500), main = "")
hist(dat$lncc, xlab = "Log CC interval", ylim = c(0,500), main = "")
```

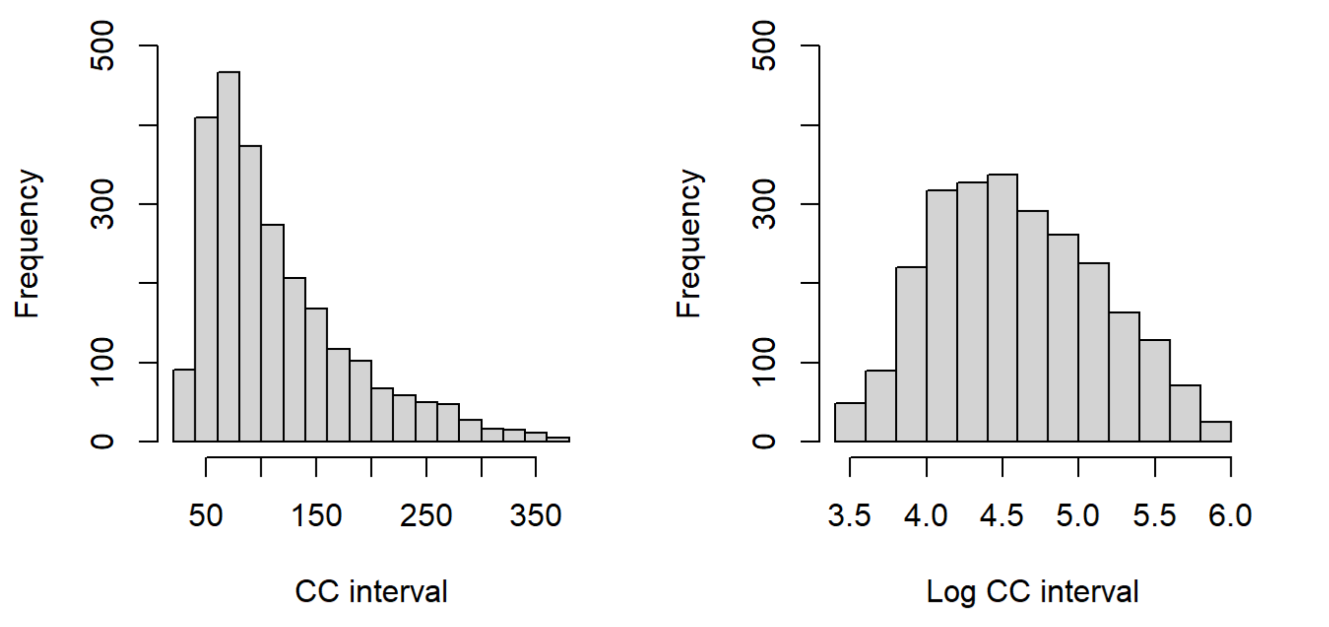

Sample size calculations:

```
# Sample size to estimate calving to conception interval assuming no clustering:
ccmu <- mean(dat$lncc); ccmu
#> [1] 4.59008
ccsigma <- sd(dat$lncc); ccsigma
#> [1] 0.5444898

# Mean log transformed calving to conception interval is 4.6; the standard deviation of log
         transformed calving to conception interval is 0.54. Calculate the number of
         lactations required, assuming the data are independent:
z <- 1.96
N <- 1000000
Vx2 <- ccsigma^2 / ccmu^2
epsilon.r <- 0.05

m1 <- ceiling((z^2 * N * Vx2) / ((1.96 * Vx2) + (N - 1) * epsilon.r^2)); m1
#> [1] 22

m1 <- epi.sssimpleestc(N = 1E06, xbar = ccmu, sigma = ccsigma, epsilon.r = epsilon.r,
         conf.level = 0.95); m1
#> [1] 22
# A total of 22 lactations are required.

# Calculate the design effect:
dat.lme01 <- lme(lncc ~ 1, random = ~1 | herd / cow, data = dat)
VarCorr(dat.lme01)
#>               Variance      StdDev
```

```r
#> herd =       pdLogChol(1)
#> (Intercept) 0.01340103   0.1157628
#> cow =        pdLogChol(1)
#> (Intercept) 0.02187348   0.1478969
#> Residual    0.26172336   0.5115890

# Calculate herd and cow level ICCs. Standard deviations of the herd, cow and lactation
         level random effect terms calculated above:
sigma3 <- 0.1157628 # [Herds]
sigma2 <- 0.1478969 # [Cows]
sigma1 <- 0.5115890 # [Lactations]

# Herd level ICC:
rho2 <- sigma3^2 / (sigma3^2 + sigma2^2 + sigma1^2); rho2
#> [1] 0.04512163

# Cow level ICC:
rho1 <- (sigma3^2 + sigma2^2) / (sigma3^2 + sigma2^2 + sigma1^2); rho1
#> [1] 0.1187703

# If we elect to sample two lactations per cow, calculate the design effect to account for
         clustering of lactations within cows:
n1 <- 2
D1 <- rho1 * (n1 - 1) + 1; D1
#> [1] 1.11877

# How many lactations are required, accounting for clustering of lactations within cow?
m2 <- ceiling(D1 * m1); m2
#> [1] 25

# How many cows are required?
n2 <- ceiling(m2 / n1); n2
#> [1] 13

# Design effect to account for clustering of cows within herds:
D2 <- 1 + (n1 * (n2 - 1) * rho2) + ((n1 - 1) * rho1); D2
#> [1] 2.201689

# How many lactations are required, accounting for clustering of lactations within cows and
         cows within herds?
m3 <- ceiling(D2 * m1); m3
#> [1] 49

# How many herds required?
n3 <- ceiling(m3 / (n1 * n2)); n3
#> [1] 2

# Total number of lactations required:
n1 * n2 * n3
#> [1] 52

# We need to sample 2 lactations from 13 cows from 2 herds (a total of 52 lactations) to
         meet the requirements of the study.
```

The required sample size assuming the data were independent was 22. The required sample size accounting for lack of independence in the data was 52, a 2.5-fold difference.

Simulation to estimate a sample size — generation of a population data set

```
# Generate a population data set. Define the number of level 3 (herd) units in the
        population. Here we generate a relatively small number of herds to reduce
        simulation times:
tn3 <- 20

# Define the number of level 2 (cow) units within each level 3 (herd) unit by taking a
        random draw from the Poisson distribution with mean equal to 150 and add 1 (to
        ensure that there are no herds of zero size):
tn2 <- rpois(n = tn3, lambda = 150) + 1

# Set the number of level 1 (lactation) units within each level 2 unit (cow) using the
        Poisson distribution. Each element of vector tn1 represents the number of lactation
        records available for each cow in each herd:
tn1 <- rpois(n = sum(tn2), lambda = 3) + 1

# Assign Level 1 (lactation) identifiers:
l1id <- seq(from = 1, to = sum(tn1), by = 1)

# Assign Level 2 (cow) identifiers to each Level 1 (lactation):
l2id <- rep(1:length(tn1), times = tn1)

# Create a population data frame listing Level 1 (lactation) and Level 2 (cow) identifiers:
pop.df <- data.frame(l1id, l2id)

# Create a lookup table to assign a Level 3 (herd) identifier to each row of the population
        data frame:
lookup <- data.frame(l3id = rep(1:tn3, times = tn2), l2id = 1:sum(tn2))
pop.df$l3id <- lookup$l3id[match(pop.df$l2id, lookup$l2id)]

# Generate a random effect term for each member of Level 1 (lactation) and create a level 1
        (lactation) random effect lookup table:
l1.lookup <- data.frame(l1id = 1:sum(tn1))
l1re.sd <- 0.511
l1.lookup$l1re <- rnorm(n = sum(tn1), mean = 0, sd = l1re.sd)

# Generate a random effect term for each member of Level 2 (cow) and create a level 2 (cow)
        random effect lookup table:
l2.lookup <- data.frame(l3id = rep(1:tn3, times = tn2), l2id = 1:sum(tn2))
l2re.sd <- 0.148
l2.lookup$l2re <- rnorm(n = sum(tn2), mean = 0, sd = l2re.sd)

# Generate a random effect term for each member of Level 3 (herd) and create a Level 3
        (herd) random effect lookup table:
l3.lookup <- data.frame(l3id = 1:tn3)
l3re.sd <- 0.116
l3.lookup$l3re <- rnorm(n = tn3, mean = 0, sd = l3re.sd)

# For each Level 1 (lactation), assign the appropriate random effect using the lookup table
        created earlier:
pop.df$l1re <- l1.lookup$l1re[match(pop.df$l1id, l1.lookup$l1id)]
```

```
# For each Level 2 (cow), assign the appropriate random effect using the lookup table
        created earlier:
pop.df$l2re <- l2.lookup$l2re[match(pop.df$l2id, l2.lookup$l2id)]

# For each Level 3 (herd), assign the appropriate random effect using the lookup table
        created earlier:
pop.df$l3re <- l3.lookup$l3re[match(pop.df$l3id, l3.lookup$l3id)]

# Assign a value for the outcome of interest to each observation:
ccmu <- mean(dat$lncc); ccmu
#> [1] 4.59008
ccsigma <- sd(dat$lncc); ccsigma
#> [1] 0.5444898
pop.df$x <- rnorm(n = sum(tn1), mean = ccmu, sd = ccsigma)

# Adjust the assigned outcome value to account fo Level 1 (lactation), Level 2 (cow) and
        Level 3 (herd) effects:
pop.df$x <- pop.df$x + pop.df$l1re + pop.df$l2re + pop.df$l3re
```

We now have a population data set where the value of the outcome variable depends on unmeasured influences varying at the lactation, cow and herd level.

Simulation to estimate a sample size — simulation of the sampling process

A series of random draws are taken from the simulated population. On how many occasions is the estimate of the population mean within the maximum tolerable error of the true population value? Here we set the maximum tolerable (relative) error to 0.05 (as we did in Box 8):

```
# Relative error:
epsilon.r <- 0.05

# Set the candidate number of herds (level 3) to be sampled:
nsl3 <- seq(from = 1, to = 10, by = 1)

# Set the candidate number of cows (level 2) to be sampled:
nsl2 <- seq(from = 1, to = 50, by = 1)

# Set the candidate number of lactations (level 1) to be sampled:
nsl1 <- 2

# Create a data frame listing the number of simulations of each sample size combination to
        run (n = 100) and the different combinations of candidate Level 3, Level 2 and
        Level 1 (herd, cow and lactation) sample sizes:
rval.df <- expand.grid(nsl3, nsl2, nsl1)
names(rval.df) <- c("nsl3", "nsl2", "nsl1")
rval.df$nsim <- 100; rval.df$nok <- NA
```

Take each Level 3, Level 2 and Level 1 sample size combination in turn. Draw the appropriate number of samples from the population and determine if the mean of the outcome variable based on the sample is within 0.05 of the true population value. Repeat 100 times. These simulations may take some time depending on the processing speed of your computer.

```r
for(i in 1:nrow(rval.df)){
  # Number of Level 3 (herds), Level 2 (cows) and Level 1 (lactations) to be sampled:
  tnsl3 <- rval.df$nsl3[i]
  tnsl2 <- rval.df$nsl2[i]
  tnsl1 <- rval.df$nsl1[i]
  ok <- rep(NA, times = rval.df$nsim[i])

  for(j in 1:rval.df$nsim[i]){
    # Sample Level 3 units (herds):
    sl3id <- sample(x = unique(pop.df$l3id), size = tnsl3, replace = FALSE)
    id <- pop.df$l3id %in% sl3id
    tpop.df <- pop.df[id,]

    # Sample Level 2 units (cows) from the selected Level 3 units (herds):
    sl2id <- by(data = tpop.df, INDICES = tpop.df$l3id,
      FUN = function(x) sample(x = unique(tpop.df$l2id), size = tnsl2, replace = FALSE))
    id <- tpop.df$l2id %in% as.numeric(unlist(sl2id))
    tpop.df <- tpop.df[id,]

    # Sample Level 1 units (lactations) from the selected Level 2 units (cows):
    sl1id <- by(data = tpop.df, INDICES = tpop.df$l2id,
      FUN = function(x) sample(x = unique(tpop.df$l1id), size = tnsl1, replace = FALSE))
    id <- tpop.df$l1id %in% as.numeric(unlist(sl1id))
    tpop.df <- tpop.df[id,]

    # Calculate the sample mean:
    emean.est <- mean(tpop.df$x)

    # Calculate the population mean:
    tmean.est <- mean(pop.df$x)
    tmean.low <- tmean.est - (tmean.est * epsilon.r)
    tmean.upp <- tmean.est + (tmean.est * epsilon.r)

    # Is the sample mean within epsilon.r of the population mean?
    ok[j] <- ifelse(emean.est >= tmean.low & emean.est <= tmean.upp, 1, 0)
    ok[j] <- ifelse(is.na(ok[j]), 0, ok[j])
  }
  rval.df$nok[i] <- sum(ok)

  # Uncomment the following line to provide a counter:
  # cat("Completed sample size combination: ", i, " of ", nrow(rval.df), "\n", sep = "")
}
```

What are the combinations of herd, cow and lactation numbers that return a calving to conception interval estimate within 5% of the true population value?

```r
head(rval.df[rval.df$nok > 95,])
#>    nsl3 nsl2 nsl1 nsim nok
#> 39    9    4    2  100  96
#> 40   10    4    2  100  99
#> 48    8    5    2  100  98
```

```
#> 49     9     5     2  100   96
#> 50    10     5     2  100   98
#> 56     6     6     2  100   97
```

Perspective plot showing the number of Level 3 (herd) and Level 2 (cow) samples on each axis and colour to indicate if the estimate of the population mean was within `epsilon.r` of the true population value:

```
# Generate a matrix of Level 3 (herd) and Level 2 (cow) sample size combinations with each
        cell of the matrix listing the proportion of samples where estimate of calving to
        conception interval was within 5% of the true population value:
z <- matrix(rval.df$nok, nrow = length(nsl3), ncol = length(nsl2))

# The argument col in persp() gives the colour in each cell, i.e. one less in each
        dimension:
cols <- z
cols <- ifelse(cols > 95, "red", "light blue")
cols <- cols[1:(dim(cols)[1] - 1),1:(dim(cols)[2] - 1)]

persp(x = nsl3, y = nsl2, z = z / 100,
   xlab = "\nNumber of herds", ylab = "\nNumber of cows", zlab = "\nProportion accepted",
     col = cols, theta = 30, phi = 30, expand = 0.8, ltheta = 100, shade = 0.6, axes = TRUE,
        ticktype = "detailed")
```

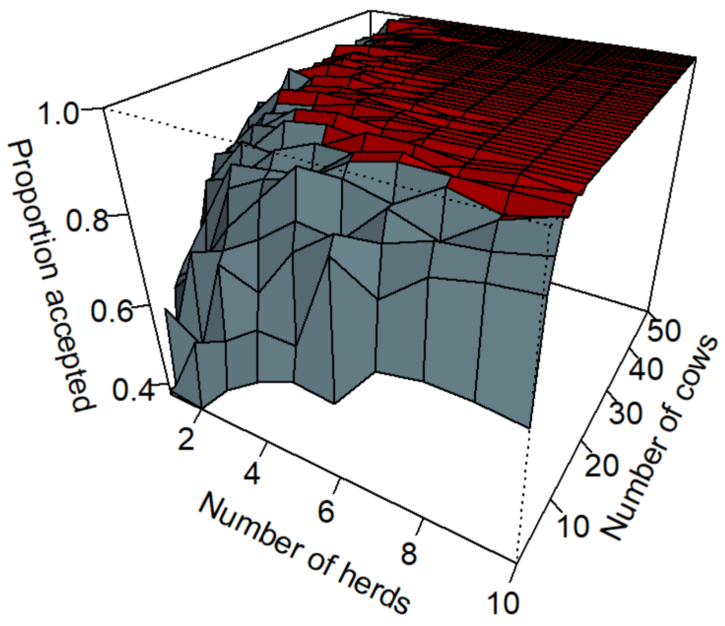

Code to reproduce Figure 2:

```
rval.df$ok <- ifelse(rval.df$nok >= 95, 1, 0)

breaks <- seq(from = 0, to = 1, length = 2)
```

```
cols <- rev(brewer.pal(n = 1, name = "Greys"))
#> Warning in brewer.pal(n = 1, name = "Greys"): minimal value for n is 3, returning
         requested palette with 3 different levels

ggplot(data = rval.df, aes(x = nsl3, y = nsl2, z = ok)) +
  theme_bw() +
  geom_raster(aes(fill = ok), hjust = 0, vjust = 0) +
  scale_fill_gradientn(colours = cols, breaks = breaks, lim = c(0,1), labels =
         c("No","Yes")) +
  scale_x_continuous(breaks = seq(from = 1, to = 10, by = 1), limits = c(1,10),
     name = "Number of herds") +
  scale_y_continuous(breaks = seq(from = 2, to = 20, by = 2), limits = c(1,20),
     name = "Number of cows") +
  theme(legend.position = c(0.80, 0.80)) +
  guides(fill = guide_legend(title = "More than 95% iterations\nwithin 5% of the\ntrue
         population value"))
#> Warning: Removed 329 rows containing missing values (geom_raster).
```

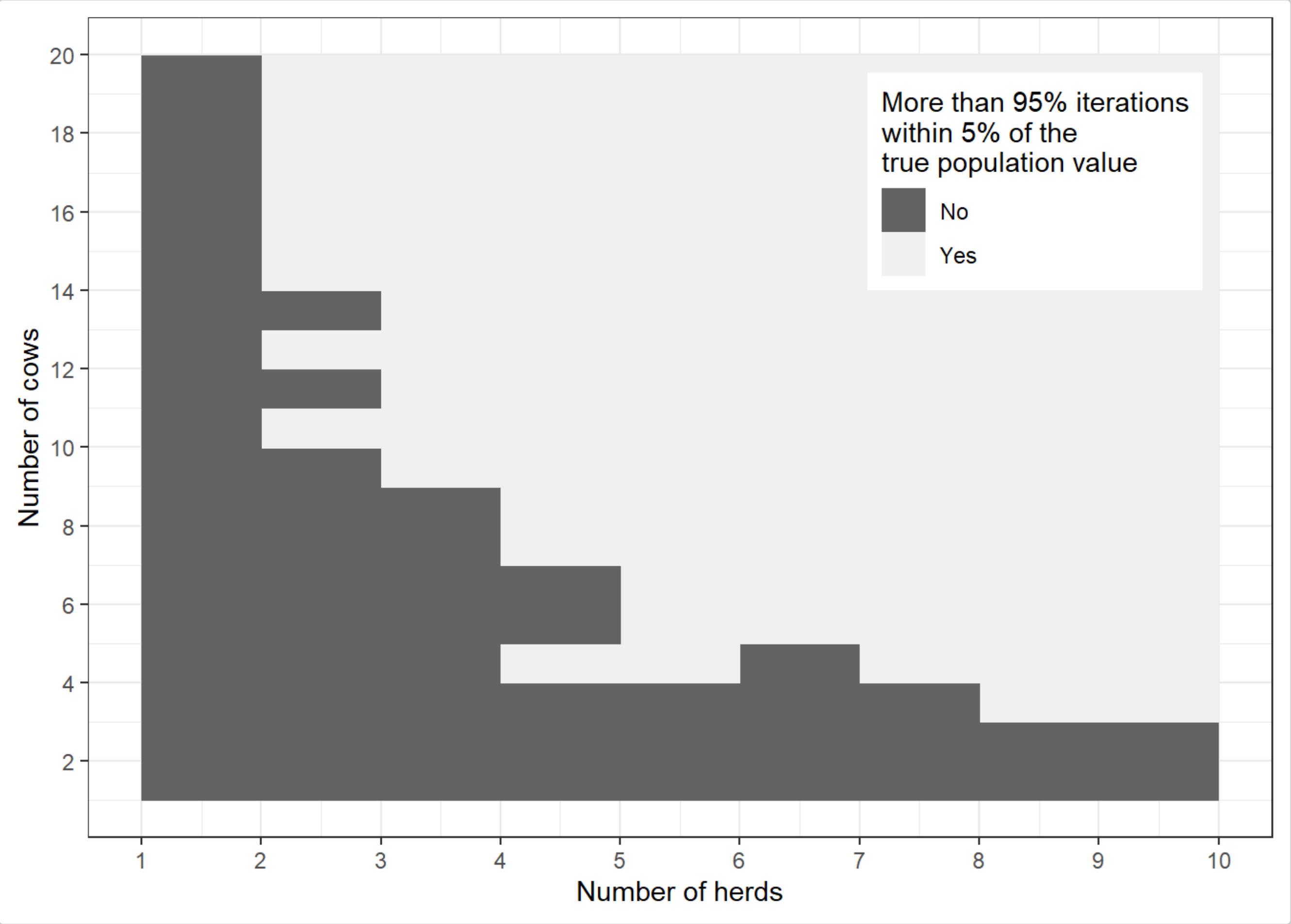

References

Dohoo, IR, E Tillard, H Stryhn, and B Faye. 2001. "The Use of Multilevel Models to Evaluate Sources of Variation in Reproductive Performance in Dairy Cattle in Reunion Island." Journal Article. *Preventive Veterinary Medicine* 50: 127–44.

Otte, JM, and ID Gumm. 1997. "Intra-Cluster Correlation Coefficients of 20 Infections Calculated from the Results of Cluster-Sample Surveys." Journal Article. *Preventive Veterinary Medicine* 31: 147–50.
